# RNA G-quadruplex (rG4) exacerbates cellular senescence by mediating ribosome pausing

**DOI:** 10.1093/procel/pwaf047

**Published:** 2025-06-12

**Authors:** Haoxian Zhou, Shu Wu, Bin Li, Rongjinlei Zhang, Ying Zou, Mibu Cao, Anhua Xu, Kewei Zheng, Qinghua Zhou, Jia Wang, Jinping Zheng, Jianhua Yang, Yuanlong Ge, Zhanyi Lin, Zhenyu Ju

**Affiliations:** Department of Cardiology, Guangdong Provincial Cardiovascular Institute, Guangdong Provincial People’s Hospital, Guangdong Academy of Medical Sciences, Guangzhou 510080, China; Key Laboratory of Regenerative Medicine of Ministry of Education, Institute of Aging and Regenerative Medicine, Department of Developmental & Regenerative Medicine, College of Life Science and Technology, Jinan University, Guangzhou 510632, China; Key Laboratory of Regenerative Medicine of Ministry of Education, Institute of Aging and Regenerative Medicine, Department of Developmental & Regenerative Medicine, College of Life Science and Technology, Jinan University, Guangzhou 510632, China; School of Life Sciences, Sun Yat-sen University, Guangzhou 510275, China; Key Laboratory of Regenerative Medicine of Ministry of Education, Institute of Aging and Regenerative Medicine, Department of Developmental & Regenerative Medicine, College of Life Science and Technology, Jinan University, Guangzhou 510632, China; Key Laboratory of Regenerative Medicine of Ministry of Education, Institute of Aging and Regenerative Medicine, Department of Developmental & Regenerative Medicine, College of Life Science and Technology, Jinan University, Guangzhou 510632, China; Department of Plastic and Reconstructive Surgery, Guangdong Second Provincial General Hospital, Guangzhou 510317, China; Key Laboratory of Regenerative Medicine of Ministry of Education, Institute of Aging and Regenerative Medicine, Department of Developmental & Regenerative Medicine, College of Life Science and Technology, Jinan University, Guangzhou 510632, China; School of Biomedical Sciences, Hunan University, Changsha 410012, China; Key Laboratory of Regenerative Medicine of Ministry of Education, Institute of Aging and Regenerative Medicine and The First Affiliated Hospital, Jinan University, Guangzhou 510632, China; Shanxi Key Laboratory of Aging Mechanism Research and Translational Applications, Changzhi Medical College, Changzhi 046012, China; Shanxi Key Laboratory of Aging Mechanism Research and Translational Applications, Changzhi Medical College, Changzhi 046012, China; School of Life Sciences, Sun Yat-sen University, Guangzhou 510275, China; Key Laboratory of Regenerative Medicine of Ministry of Education, Institute of Aging and Regenerative Medicine, Department of Developmental & Regenerative Medicine, College of Life Science and Technology, Jinan University, Guangzhou 510632, China; Department of Cardiology, Guangdong Provincial Cardiovascular Institute, Guangdong Provincial People’s Hospital, Guangdong Academy of Medical Sciences, Guangzhou 510080, China; Key Laboratory of Regenerative Medicine of Ministry of Education, Institute of Aging and Regenerative Medicine, Department of Developmental & Regenerative Medicine, College of Life Science and Technology, Jinan University, Guangzhou 510632, China

**Keywords:** cellular senescence, ribosme pausing, RNA G-quadruplex

## Abstract

Loss of protein homeostasis is a hallmark of cellular senescence, and ribosome pausing plays a crucial role in the collapse of proteostasis. However, our understanding of ribosome pausing in senescent cells remains limited. In this study, we utilized ribosome profiling and G-quadruplex RNA immunoprecipitation sequencing techniques to explore the impact of RNA G-quadruplex (rG4) on the translation efficiency in senescent cells. Our results revealed a reduction in the translation efficiency of rG4-rich genes in senescent cells and demonstrated that rG4 structures within coding sequence can impede translation both *in vivo* and *in vitro*. Moreover, we observed a significant increase in the abundance of rG4 structures in senescent cells, and the stabilization of the rG4 structures further exacerbated cellular senescence. Mechanistically, the RNA helicase DHX9 functions as a key regulator of rG4 abundance, and its reduced expression in senescent cells contributing to increased ribosome pausing. Additionally, we also observed an increased abundance of rG4, an imbalance in protein homeostasis, and reduced DHX9 expression in aged mice. In summary, our findings reveal a novel biological role for rG4 and DHX9 in the regulation of translation and proteostasis, which may have implications for delaying cellular senescence and the aging process.

## Introduction

Cellular senescence plays a significant role in the aging of organs and organisms, as the accumulation of senescent cells is closely associated with the aging process and the degeneration of various tissues and organs (Gorgoulis et al., 2019). Furthermore, the imbalance in proteostasis, a fundamental characteristic of cellular senescence, is evident in numerous age-related diseases (Lopez-Otin et al., 2013; Montague-Cardoso, 2021; Rubinsztein et al., 2011; Salminen et al., 2012). Neurodegenerative disorders, for instance, are characterized by the abnormal aggregation of proteins (Galluzzi et al., 2018; Godin et al., 2016; Wilson et al., 2023). Protein synthesis and degradation are subject to precise regulation within cellular contexts. When errors occur during these processes, cellular mechanisms responsible for maintaining protein quality control are activated. These mechanisms include the unfolded protein response (UPR; Senft and Ronai, 2015), the ubiquitin-proteasome system (Pohl and Dikic, 2019), and the autophagy-lysosome pathway (Sha et al., 2017). The principal aim of these response mechanisms is to prevent the excessive accumulation of misfolded or unassembled peptides by facilitating the degradation of defective proteins through the ubiquitin-proteasome system and the autophagy-lysosome pathway.

Additionally, the maintenance of protein homeostasis is facilitated by the active engagement of ribosome-associated quality control through ribosome pausing (Brandman et al., 2012; Howard and Frost, 2021; Shao et al., 2015). When ribosomes encounter stalling during the translation, ZNF598 identifies the stalled ribosomes and facilitates the activation of eIF2α (Darnell et al., 2018; Shao et al., 2015). Consequently, a cascade of ribosomal proteins dissociation factors is recruited to facilitate the degradation of truncated peptides and prevent the aggregation of incorrect peptide segments. This process effectively preserves cellular protein homeostasis. Ribosome pausing is associated with various factors including the cell cycle (VanInsberghe et al., 2021), leucine limitation (Darnell et al., 2018), and aging (Stein et al., 2022). Previous studies have reported that ribosomes tend to pause at proline, glycine, and aspartic acid codon motifs (Han et al., 2020), with some of these motifs being enriched in guanine codons. In prokaryotes, ribosomes stall at Shine–Dalgarno sequences (Li et al., 2012), which are composed of adenine and guanine bases. This observation suggests that ribosomes may pause at sequences enriched in guanine. However, the precise underlying mechanism by which aging induces ribosome stalling remains unclear.

G-quadruplexes represent non-canonical secondary structures of DNA or RNA that primarily emerge in guanine-rich regions, displaying diverse topological configurations, including parallel, anti-parallel, and hybrid conformations (Yuan et al., 2020). Although the structure and function of DNA G-quadruplexes have been comprehensively studied, our understanding of RNA G-quadruplexes (rG4) and their functions remains relatively limited. Putative rG4s are ubiquitously present in both coding and non-coding regions of pre-mRNAs and mRNAs, including introns, 5′- and 3′-UTRs (Fay et al., 2017; Kharel et al., 2023). Previous studies have demonstrated that rG4 located in the 5′UTR can impede protein translation, whereas those present in ribosomal genes can suppress protein expression levels (Murat et al., 2018). However, recent research has shown that 5′UTR rG4s can enhance translation in *Escherichia coli* (Lee et al., 2024). The prevalence of these G4s in mRNA regions associated with regulatory functions suggests their involvement in the regulation of mRNA metabolism.

Here, we provide evidence that ribosome pausing is prevalent in the G-rich regions of mRNA, particularly in senescent cells with higher levels of rG4. Furthermore, our findings demonstrate that the increased localization of rG4 in mRNA leads to enhanced ribosome pausing, which in turn contributes to the disruption of proteostasis and the progression of cellular senescence. Additionally, DHX9 acts as a critical regulator of rG4 abundance, whereby its diminished expression in senescent cells exacerbates ribosome pausing. Moreover, we also observe a similar regulatory mechanism in mouse TFB (tail-tip fibroblast) cells and highly aged tissues of older mice, thereby suggesting a broader regulatory framework. Consequently, our findings reveal that rG4 and DHX9 serve as novel regulators of translation and proteostasis that constitutes a critical mechanism underlying cellular senescence and aging.

## Results

### Ribosome pausing reduces translation efficiency and is enriched in G-rich regions of mRNA in senescent cells

**Figure 1.**
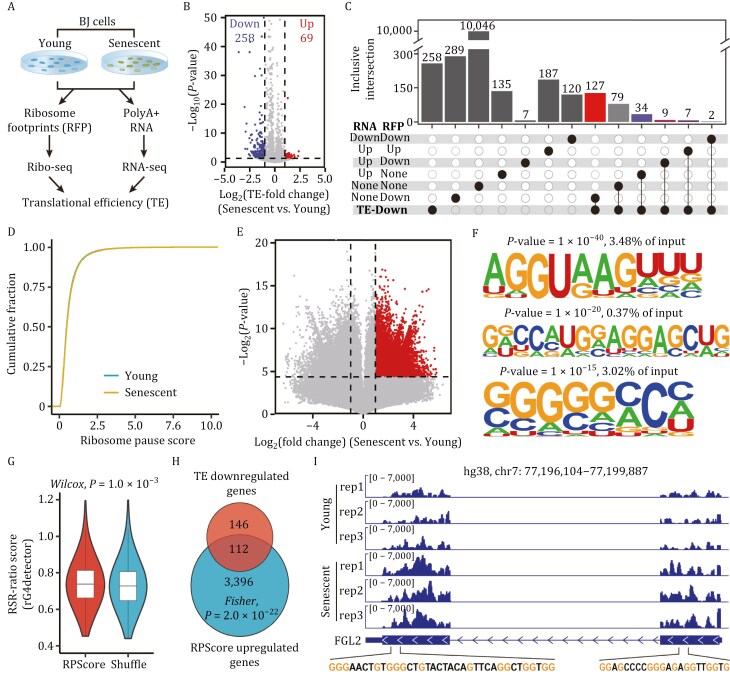
Ribosome pausing-reduced TE is enriched in G-rich regions in senescent cells. (A) Schema illustrating the workflow for analyzing TE in young (proliferative stage) and replication senescent cells. (B) Volcano plot illustrating changes in translational efficiency between young and senescent BJ cells, with cutoffs set at *P* < 0.05 and |log2TE-fold change| ≥ 1. (C) Intersection analysis of genes with different patterns of change in transcriptome and translatome and genes with downregulated TE. The intersection analysis among these uses the Inclusive intersection mode in ComplexUpSet. (D) Cumulative frequency histogram of ribosome pause scores in the CDS of senescent and young BJ cells. (E) Relative ribosome pausing during cellular senescence. The colored points indicate codon positions with significantly increased pausing (fold change ≥ 2, *P* value < 0.05), all other translatome positions in gray. Statistical analysis was performed using a two-sided Wilcoxon test. (F) Enriched motifs identified in sequences located a 20-nt region downstream of RPScore upregulated site using the Homer software. The frequency of occurrence for each motif across all input sequences has also been included in the figures. (G) Violin plots show RSR-ratio score of the sequences spanning 20 nucleotides downstream of the downregulated RPScore sites and or shuffled sequences. Box plots show minimum value, first quartile, median, third quartile, and maximum value. The *P*-value between the two categories were determined by the Mann–Whitney–Wilcoxon test. (H) Analysis of the intersection between RPScore-upregulated genes and genes exhibiting reduced TE. The *P*-value was calculated by Fisher’s exact test. (I) Integrative Genomics Viewer (IGV) depicting senescent cells ribosome occupies sequence is comprise guanine enrich region.

The collapse of proteostasis is a significant characteristic of cellular senescence (Lopez-Otin et al., 2013). To elucidate the underlying physiological processes contributing to this phenomenon, we conducted RNA-seq and Ribo-seq analyses on both young (proliferative stage) and replicative senescent BJ fibroblast cells (Figs. 1A and S1A–E). Consistent with previous observations (Stein et al., 2022), we identified a marked reduction in translation initiation in senescent cells (Fig. S1F). We further investigated the alterations in mRNA translation efficiency (TE) between young and senescent cells. TE is defined as the ratio of ribosome footprints (RFPs) to mRNA abundance, as measured by Ribo-seq and RNA-seq, respectively. We calculated TE using the Xtail algorithm (Xiao et al., 2016). Remarkably, the number of genes exhibiting reduced TE is approximately four times greater than the count of upregulated genes (Fig. 1B). To further investigate the potential mechanism contributing to TE dysregulation, we conducted an in-depth analysis of the transcriptome and translatome change patterns of genes with downregulated TE (Figs. 1C and S2A). Our analysis revealed that the greatest number of genes displaying changes is exclusively observed at the translational level, specifically in the “RNA none-RFP down” category (Fig. 1C). Additionally, it is noteworthy that ~50% of the genes showing a reduction in TE overlapped with these alterations (Fig. S2B).

To elucidate the factors contributing to the reduced TE, we calculated a ribosome pause score (RPScore) for each position within a coding sequence (CDS) relative to the entire open reading frame. The cumulative distribution of RPScores across the transcriptome revealed no global senescence-related changes (Fig. 1D). With the exception of methionine, there were negligible variations in the relative ribosome density for other amino acids in the context of senescence (Fig. S1G). This suggests that senescence does not induce a systemic alteration in overall elongation pausing. However, considering the possibility that senescence might induce specific changes in translation elongation, we proceeded to extract sequences spanning 20 nucleotides downstream of the downregulated RPScore sites (Fig. 1E) in senescent cells for subsequent motif enrichment analysis. Our findings revealed that the ribosome tended to pause before guanine-rich motifs (Figs. 1F and S2C). When analyzed with rG4 detector, an RNA G-quadruplex predictor (Turner et al., 2022), the sequences spanning 20 nucleotides downstream of the downregulated RPScore sites exhibited significantly higher RSR-ratio scores compared with shuffled sequences (Fig. 1G). Moreover, ~43.4% (112 out of 258) of the genes displaying translational downregulation also exhibited a concurrent downregulation of RPScore (Fig. 1H). Additionally, we discovered that *FURIN*, one of the key endoprotease, exhibited decreased TE in senescent cells and contained regions enriched with guanine nucleotides (Fig. 1I). Overall, ribosome pausing diminishes TE within G-rich regions in senescent cells.

### RNA G-quadruplex signal is increased in senescent cells and hinders translation

**Figure 2.**
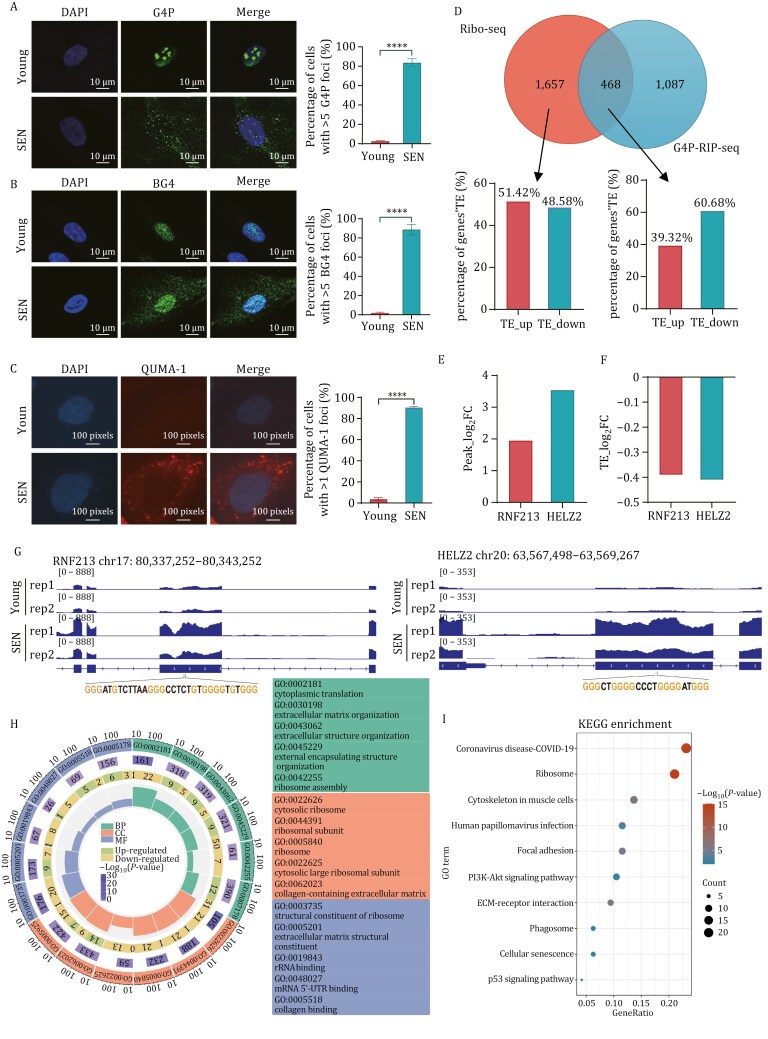
RNA G-quadruplex signal is increased in senescent cells and hinders translation. (A) Cytoplasm G4P signal was increased in young and replication senescent G4P-cells. G4P was induced by DOX and IF was performed with Flag antibody. Cells contain more than one cytoplasm G4P foci were calculated (*n* ≥ 100 cells). All values are mean ± SEM of more than three independent experiments. (B) Cytoplasm BG4 signal was increased in replication senescent cells. IF was performed with BG4 and Flag antibody. Cells contain more than one cytoplasm BG4 foci were calculated (*n* ≥ 100 cells). All values are mean ± SEM of more than three independent experiments. (C) rG4 was increased in replication senescent cells. Imaging of replication senescent cells stained with QUMA-1 (2 μmol/L) for 20 min at 37°C. Cells contain more than one cytoplasm QUMA-1 foci were calculated (*n* ≥ 100 cells). All values are mean ± SEM of more than three independent experiments. (D) Most intersection genes between Ribo-seq and G4P-RIP-seq have decreased TE in senescent cell. (E) G4P-RIP-seq enrich high-fold change genes in senescent cell. (F) According to (D), the protein TE of rG4-enriched genes is decreased. (G) IGV depicting G4P occupies peak according to (D) in senescent cell. (H) GO functional enrichment analysis of differentially enrich genes between G4P-senescent and G4P-young cells. (I) KEGG pathway enrichment analysis from differentially enriches genes between G4P-senescent and G4P-young cells.

G-quadruplexes are prone to form, we propose that they influence translation in senescent cells. To test this hypothesis, we conducted experiments to empirically investigate their effects. Following this, we investigated the prevalence of rG4 in senescent cells. In young cells, the G-quadruplex binding protein G4P (Zheng et al., 2020) and BG4 (Lee et al., 2024) were predominantly localized within the nucleus. However, in both replication-induced and damage-induced senescent cells, the majority of the G4P and BG4 foci were observed in the cytoplasm, indicating an increased presence of rG4 in senescent cells (Figs. 2A, 2B, and S3A–G). Given that G4P and BG4 are not exclusively specific to rG4, we employed a rG4-specific probe, QUMA-1 (Chen et al., 2018), for confirmation. Consistently, rG4 levels were found to be significantly increased in both replication- and damage-induced senescent cells (Figs. 2C, S3H and S3I). These findings provide evidence that rG4 levels are elevated in senescent cells.

Next, we established a stable BJ fibroblast cell line expressing the G-quadruplex binding protein G4P and collected both young and senescent cells for RIP-seq analysis (Fig. S4A–C). By integrating these data with the Ribo-seq data, we found that 60.68% of the genes in the overlapping portion between Ribo-seq and G4P-RIP-seq exhibited decreased TE in senescent cells. In contrast, this percentage fell to 48.58% in the non-overlapping sets (Figs. 2D and S4E). Moreover, several genes showing significant fold changes and enrichment in G4P senescent cells also exhibited decreased TE, including RNF213 and HELZ2, both of which contain potential rG4 sequence in their CDS region (Fig. 2E–G). Furthermore, we conducted KEGG and GO analyses on the differentially expressed genes identified from young and senescent G4P-RIP-seq and found that the majority of rG4-enriched genes are implicated in ribosome-related biological pathways (Fig. 2H and 2I). This observation highlights the significant role of rG4 in translation.

### RNA G-quadruplexs exacerbate ribosome pausing leading to translation impairment

**Figure 3.**
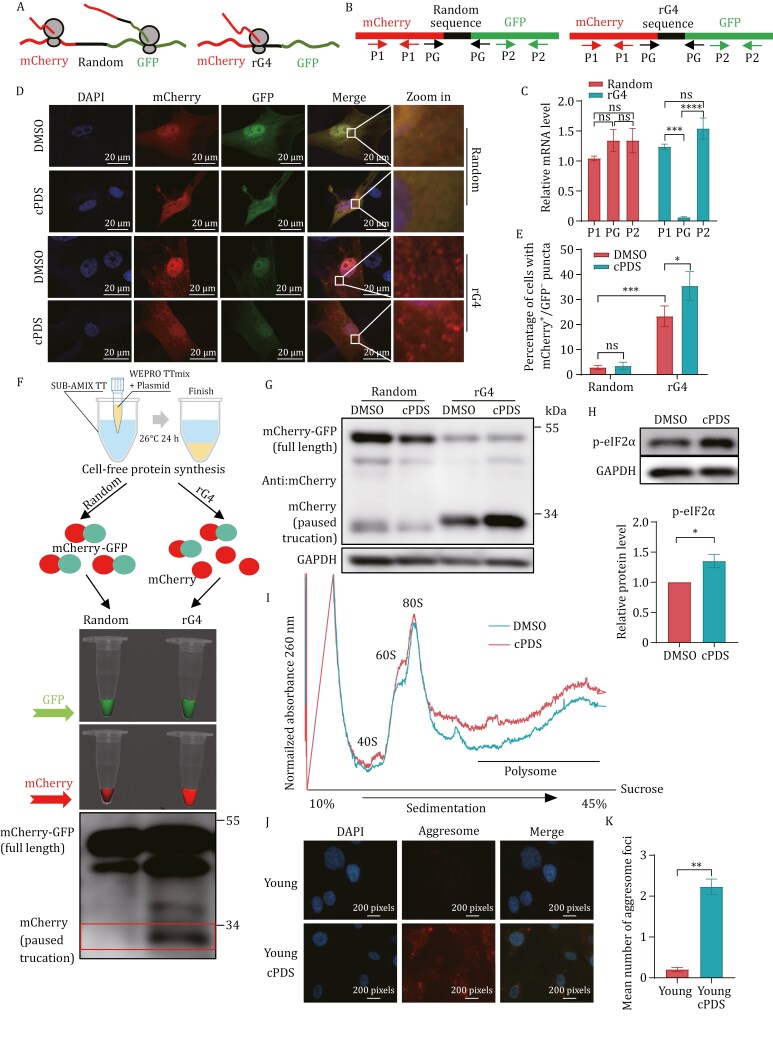
RNA G-quadruplex exacerbates ribosome pausing leading to translation impairment. (A) Schematic diagram of the dual fluorescence reporter. (B) Schematic diagram of the primer design. (C) Random and rG4 sequences not affect the dual fluorescence reporter transcription. mRNA levels were quantified by qPCR. (D) Fluorescence microscopy and mCherry^+^/GFP^−^ puncta formation in BJ fibroblast cells harboring the dual fluorescence reporter. BJ cells were transfected with dual fluorescence reporter for 72 h and treated with cPDS (2 μmol/L). (E) Quantification of (D). Cells contain mCherry^+^/GFP^−^ puncta were calculated (*n* ≥ 50 cells). All values are mean ± SEM of more than three independent experiments. (F) CFS. According to (A), use CFS to generate mCherry-random-GFP fusion protein or mCherry-rG4-GFP fusion protein *in vitro*. (G) Immunoblot of dual fluorescence reporter in 293T cells. 293T cells were transfected with dual fluorescence reporter for 72 h and treated with cPDS (2 μmol/L). Western was performed with indicated antibodies. Truncated and full-length products are noted. *n* = 3 biological replicates with representative example shown. (H) Immunoblot analysis of indicated proteins in BJ cells after treated with cPDS (2 μmol/L) for 48 h (*n* = 3). (I) Polysomes increased following cPDS treatment. Shown is a representative UV trace of polyribosome gradients from presenescent BJ cell (4 PD prior to senescence) lysates, either untreated or treated with cPDS. *n* = 2 biological replicates with representative example shown. (J) Representative aggresome staining images of young and cPDS treatment cells. Aggresome increased in cPDS treatment cells. (K) Quantification of (J). Cells contain more than one aggresome foci were calculated (*n* ≥ 100 cells). All values are mean ± SEM of more than three independent experiments.

As previously discussed, the utilization of omics sequencing analysis has provided evidence that rG4 hinders the process of translation. To further substantiate this hypothesis at the molecular level, we developed “mCherry-GFP” dual fluorescence reporter system (Fig. 3A). The mCherry-GFP fusion protein was engineered with either a random sequence or a rG4 sequence, neither of which impeded the transcription (Fig. 3B and 3C). If ribosomes encounter a pause at the rG4 sequence, it would lead to the formation of mCherry-positive but GFP-negative puncta. Subsequently, the reporter system was transfected into BJ cells, resulting in a higher abundance of mCherry^+^/GFP^−^ puncta in the rG4 group compared with the random group. Significantly, the phenomenon observed in the rG4 group was notably augmented by the application of cPDS, a compound known for stabilizing rG4 structures in cells (Kharel et al., 2023) (Fig. 3D–E). In addition to engineering the artificial rG4 sequence into the “mCherry-GFP” dual fluorescence reporter system, we also inserted actual G4 and rG4 sequences into the reporter system as the sense strand, which are identified from promoter or CDS regions and allow transcription of the “mCherry-rG4-GFP” mRNA. Consistent with the results for the artificial rG4 sequences, all actual rG4 sequences also hindered ribosome elongation, leading to the production of truncated mCherry proteins (Fig. S5A and S5B).

Moreover, we confirmed the inhibition of translation by rG4 through *in vitro* cell-free protein synthesis (CFS). We observed a sustained increase in mCherry protein production specifically within the rG4 group, indicating a pause in translation prior to GFP synthesis (Fig. 3F). To substantiate this finding, we performed western blot analysis, which confirmed an increased production of truncated mCherry protein in the rG4 group (Fig. 3G). Ribosome pausing can trigger ribosome collision and activate eIF2α, JNK, and p38 MAPK (Darnell et al., 2018). Consistent with this, treatment with cPDS led to the phosphorylation of eIF2α (Fig. 3H). To determine whether rG4 sequences contribute to ribosome collisions, we employed cPDS to stabilize rG4 and subsequently conducted polysome profiling. The results showed a significant increase in polysome levels following cPDS treatment (Fig. 3I). Furthermore, in addition to the activation of eIF2α, total translation would be inhibited after cPDS treatment. To validate this assumption, we performed a puromycin incorporation assay, which yielded results consistent with eIF2α activation (Fig. S5C). Moreover, ribosome pausing also contributes to the disruption of proteostasis. Consequently, we examined the aggresome and noted a significant increase in cPDS-treated cells (Fig. 3J–K). These finding provide strong evidence that rG4 exacerbates ribosome pausing.

### Stabilization of RNA G-quadruplexs accelerates cellular senescence

**Figure 4.**
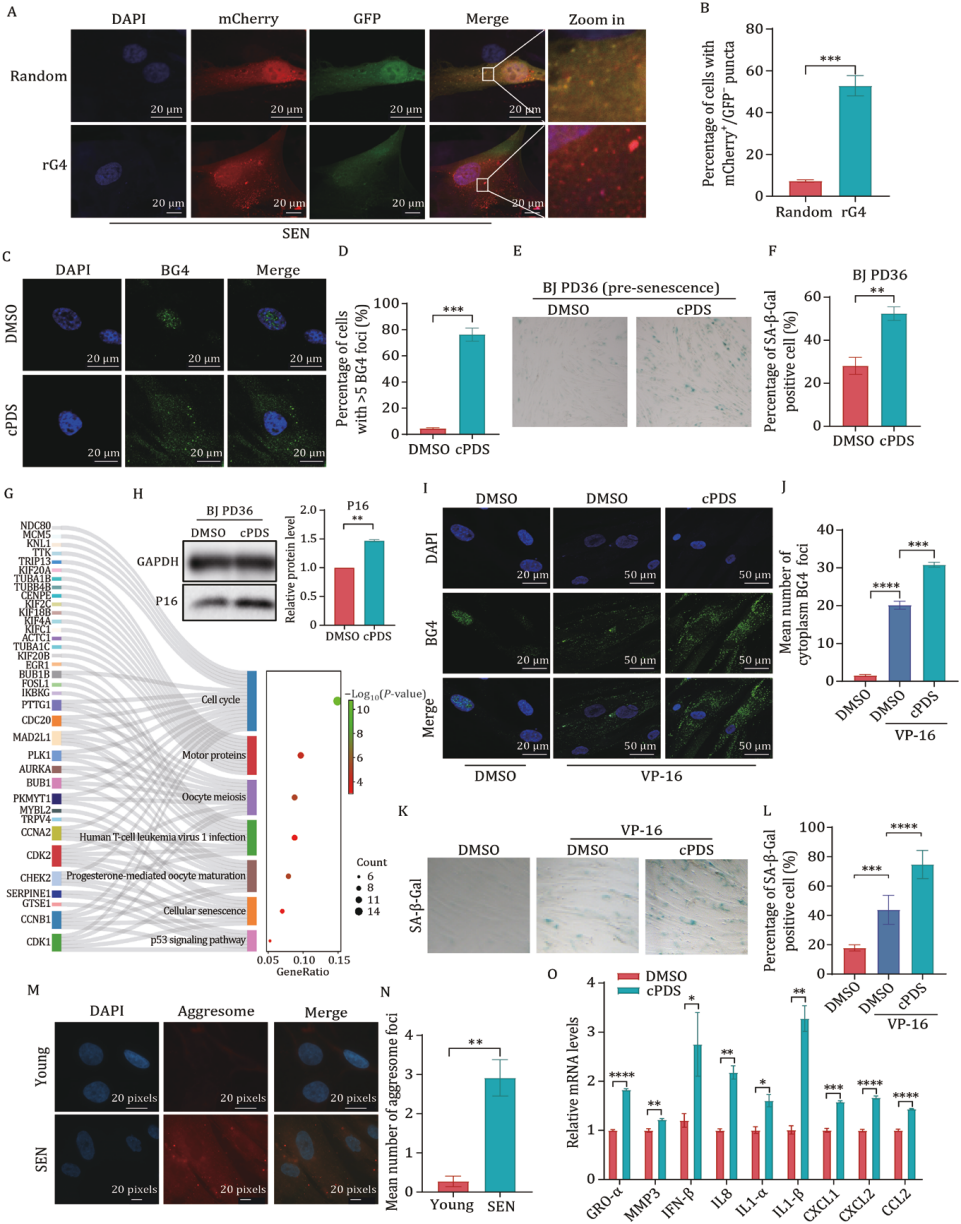
Stabilization of RNA G-quadruplex accelerates cellular senescence. (A) Fluorescence microscopy and mCherry^+^/GFP^−^ puncta formation in young and senescent BJ fibroblast cells harboring the dual fluorescence reporter. Young and senescent BJ cells were transfected with dual fluorescence reporter for 72 h. (B) Quantification of (A). Cells contain mCherry^+^/GFP^−^ puncta were calculated (*n* ≥ 50 cells). All values are mean ± SEM of more than three independent experiments. (C) cPDS increased rG4 in presenescent cells. PD36 BJ cells were treated with cPDS (2 μmol/L) for 48 h. IF was performed with BG4 and Flag antibodies. (D) Quantification of (C). Cells contain more than one cytoplasm BG4 foci were calculated (*n* ≥ 50 cells). All values are mean ± SEM of more than three independent experiments. (E) Stabilization of rG4 increased SA-β-Gal positive cells. (F) Quantification of (E). SA-β-Gal positive cells were calculated (*n* ≥ 100 cells). All values are mean ± SEM of more than three independent experiments. (G) KEGG pathway enrichment analysis of differentially enrich genes between DMSO and cPDS treatment BJ cells. (H) Immunoblot analysis of indicated proteins in presenescent BJ cells after treated with cPDS (2 μmol/L) for 48 h (*n* = 3). (I) cPDS increased rG4 in damage-induced senescent cells. BJ cells were treated with VP-16 (40 μmol/L) for 24 h and then released for 48 h to induce cellular senescence and treated with cPDS (2 μmol/L) for 48 h. IF was performed with BG4 and Flag antibodies. (J) Quantification of (I). Cells contain more than one cytoplasm BG4 foci were calculated (*n* ≥ 50 cells). All values are mean ± SEM of more than three independent experiments. (K) Stabilization of rG4 accelerates cellular senescence. BJ cells were treated according with (G) and stained by SA-β-Gal. (L) Quantification of (K). SA-β-Gal positive cells were calculated (*n* ≥ 100 cells). All values are mea ± SEM of more than three independent experiments. (M) Representative aggresome staining images of young and senescent cells. Aggresome increased in senescent cells. (N) Quantification of (M). Cells contain more than one aggresome foci were calculated (*n* ≥ 100 cells). All values are mean ± SEM of more than three independent experiments. (O) SASPs were increased after cPDS treatment in senescent cells. Detection of mRNA levels of SASPs treated with cPDS (2 μmol/L) for 48 h in senescent cells. mRNA levels were quantified by qPCR.

Proteostasis imbalance is a key characteristic of cellular senescence. To investigate whether ribosome pausing contribute to the cellular senescence, we employed the “mCherry-GFP” dual fluorescence reporter system and observed a higher abundance of mCherry^+^/GFP^−^ puncta in the rG4 group in the senescent cells, suggesting that rG4 caused ribosome pausing in senescent cells (Fig. 4A and 4B). Next, in order to prove whether stabilizing rG4 affects the senescence process, presenescent BJ fibroblast cells were subjected to cPDS treatment. SA-β-Gal staining, a widely recognized marker of senescent cells, was performed to assess the effects. The results revealed that cPDS treatment led to a significant increase in the proportion of SA-β-Gal-positive cells (Fig. 4C–F). Additionally, we conducted RNA-seq analyses on both control and cPDS-treated BJ fibroblast cells (Fig. S6) and found that the upregulated genes were notably enriched in cell cycle and cellular senescence processes (Fig. 4G). This suggests that cPDS treatment accelerates cellular senescence. Subsequently, we employed western blot analysis to evaluate the levels of P16, a well-established marker of senescence. It was observed that the levels of p16 increased after cPDS treatment (Fig. 4H).

To further evaluate the senescence process, we treated the cells with etoposide (VP-16) to induce cellular senescence (damage-induce senescence) (Bang et al., 2019) in combination with cPDS treatment and observed consistent outcomes. This was evidenced by an increased presence of rG4 signals and SA-β-Gal-positive cells (Fig. 4I–L), which aligns with the results observed in the presenescent cells. Furthermore, we investigated the aggresome and identified a significant augmentation in senescent cells, indicating the disruption of proteostasis (Fig. 4M and 4N). In addition to proteostasis, the senescence-associated secretory phenotype (SASP) is another hallmark of cellular senescence, was detected by qPCR which showed a notable increase in the group treated with cPDS (Fig. 4O). These findings provide substantial empirical support for the notion that enhancing the stability of the rG4 structure accelerates cellular senescence.

### DHX9 deficiency facilitates RNA G-quadruplex stability and promotes cellular senescence

**Figure 5.**
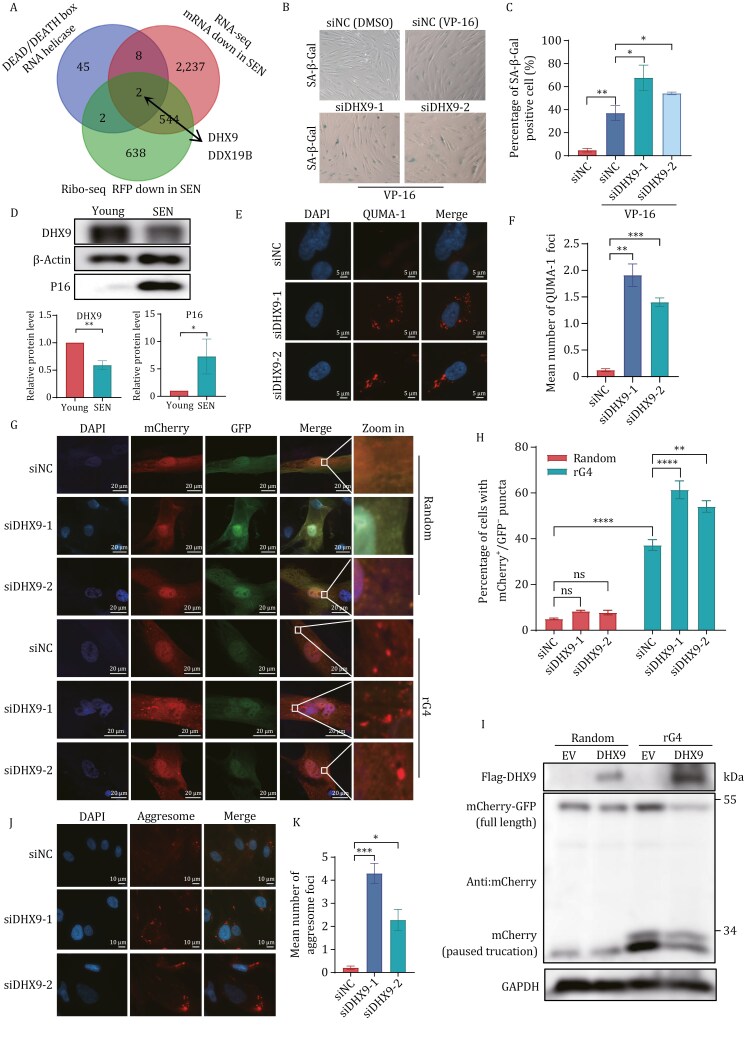
DHX9 deficiency facilitates RNA G-quadruplex stability and promotes cellular senescence. (A) Veen diagram representing the overlapping genes of DEAD box and DEATH box RNA helicase identified through RNA-seq and Ribo-seq. Only DHX9 and DDX19B showed decreased mRNA and translation levels in senescent cells. (B) DHX9 deficiency accelerates cellular senescence. BJ cells were treated with VP-16 (40 μmol/L, 24 h) and transfected with siRNAs (72 h), then stained by SA-β-Gal. (C) Quantification of (B). SA-β-Gal positive cells were calculated (*n* ≥ 100 cells). All values are mean ± SEM of more than three independent experiments. (D) Immunoblot analysis of indicated proteins in young and senescent BJ cells. (E) rG4 was increased in DHX9 deficiency cells. BJ cells were transfected with siRNAs for 72 h. Imaging of DHX9 deficiency cells stained with QUMA-1 (2 μmol/L) for 20 min at 37°C. (F) Quantification of (E). Cells contain more than one cytoplasm QUMA-1 foci were calculated (*n* ≥ 100 cells). All values are mean ± SEM of more than three independent experiments. (G) Fluorescence microscopy and mCherry^+^/GFP^−^ puncta formation in DHX9 deficiency BJ fibroblast cells harboring the dual fluorescence reporter. BJ cells were transfected with dual fluorescence reporter and siRNAs for 72 h. (H) Quantification of (G). Cells contain mCherry^+^/GFP^−^ puncta were calculated (*n* ≥ 100 cells). All values are mean ± SEM of more than three independent experiments. (I) Immunoblot of dual fluorescence reporter in 293T cells, and 293T cells were transfected with dual fluorescence reporter and Flag-DHX9 for 72 h. Western blot was performed with indicated antibodies. Truncated and full-length products are noted. *n *= 3 biological replicates with representative example shown. (J) Representative aggresome staining images of DHX9 deficiency BJ cells. Aggresome increased in DHX9 deficiency BJ cells. BJ cells were transfected with siRNAs for 72 h. (K) Quantification of (J). Cells contain more than one aggresome foci were calculated (*n* ≥ 100 cells). All values are mean ± SEM of more than three independent experiments.

To elucidate the underlying mechanism responsible for the observed increase in rG4 structures in senescent cells, we conducted a screening of the helicases that bind and unwind rG4 structures by integrating RNA-seq and Ribo-seq analysis. Our findings revealed that only DHX9 and DDX19B exhibited decreased levels of both transcription and translation in senescent cells (Fig. 5A). In addition, our results demonstrated that the deficiency of DHX9 led to a significantly higher increase in the proportion of SA-β-Gal-positive cells (Figs. 5B, 5C, S7A and S7B). This suggests that DHX9 serves as the primary helicase involved in regulating rG4 structures, which also diminished in senescent cells (Fig. 5D). Subsequently, QUMA-1 was performed to detect rG4 levels in DHX9-deficient cells, revealing a notable increase in the abundance of rG4 structures (Fig. 5E and 5F). Moreover, we conducted simultaneous transfection of the “mCherry-GFP” dual fluorescence reporter system and DHX9 siRNA to explore the potential role of DHX9 in regulating translation. The results demonstrated that DHX9 deficiency had no impact on the random group but resulted in an increased number of mCherry^+^/GFP^−^ puncta and truncated mCherry protein in the rG4 group (Figs. 5G, 5H, S7C and S7D). This suggests an increased frequency of ribosome pausing in DHX9-deficient cells. Conversely, overexpression of DHX9 attenuated the production of truncated mCherry protein and the proportion of SA-β-Gal-positive cells (Figs. 5I and S7E–G). Furthermore, the presence of aggresome was observed in DHX9-deficient cells, with a significant increase observed following DHX9 knockdown (Fig. 5J and 5K). These findings highlight the crucial role of DHX9 in the translation of senescent cells, specifically through its function in the unwinding of rG4 structures.

### Disruption of proteostasis accompanied by reduced DHX9 level and increased rG4 abundance in the liver and lung of aged mice

**Figure 6.**
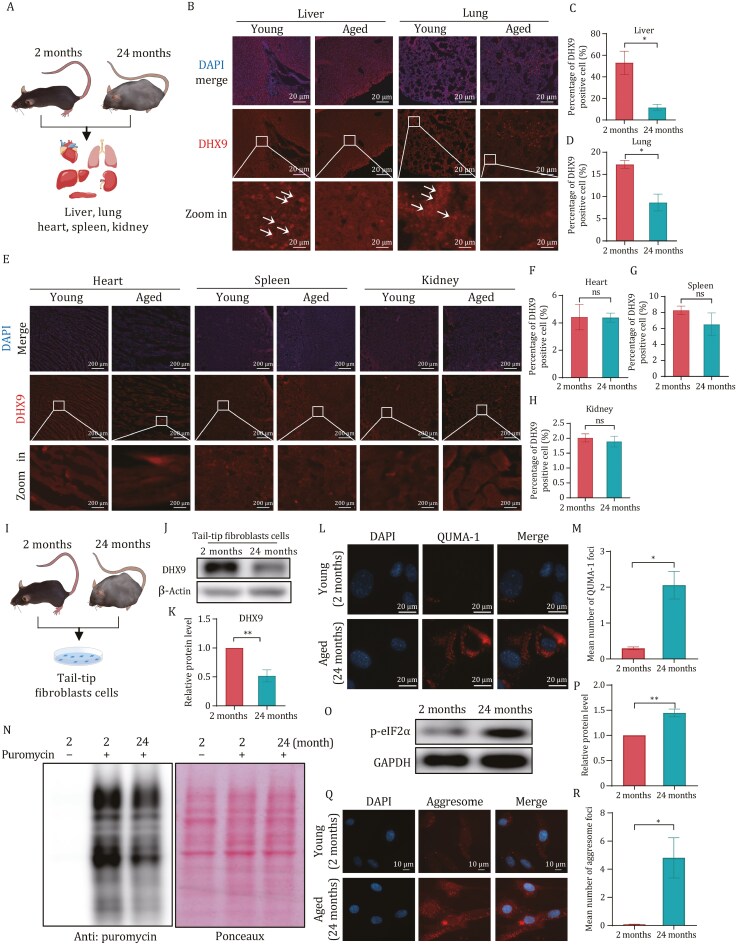
Disruption of proteostasis accompanied by reduced DHX9 level and increased rG4 abundance in aged mice. (A) Schematic diagram of organs isolation. (B) Immunohistochemical analysis of DHX9 expression in Liver and Lung. (C and D) Quantification of (B). DHX9 positive cells were calculated. All values are mean ± SEM of more than three mice. (E) Immunohistochemical analysis of DHX9 expression in Heart, Spleen, and Kidney. (F–H) Quantification of (E). DHX9 positive cells were calculated. All values are mean ± SEM of more than three mice. (I) Schematic diagram of Tail-tip fibroblast cells isolation. (J) Immunoblot analysis of indicated proteins in young and aged Tail-tip fibroblast cells. (K) Quantification of (J). Relative protein level of DHX9. (L) rG4 was increased in aged mice Tail-tip fibroblast cells. Imaging of Tail-tip fibroblast cells stained with QUMA-1 (2 μmol/L) for 20 min at 37°C. (M) Quantification of (L). Cells contain more than one cytoplasm QUMA-1 foci were calculated (*n* ≥ 100 cells). All values are mean ± SEM of more than three independent experiments. (N) Puromycin incorporation assay using TFB cells shows reduced Puromycin incorporation in 24 months TFB. The right panel is the Ponceau-S stained membrane for the same blot. *n* = 3 biological replicates with representative example shown. (O) Immunblot analysis of indicated proteins in 2-month and 24-month TFB (*n* = 3). (P) Quantification of relative protein level of p-eIF2α. (Q) Representative aggresome staining images of young and aged mice Tail-tip fibroblast cells. Aggresome increased in aged mice Tail-tip fibroblast cells. (R) Quantification of (Q). Cells contain more than one aggresome foci were calculated (*n* ≥ 100 cells). All values are mean ± SEM of more than three independent experiments.

To establish the physiological relevance of this phenomenon, we examined its presence *in vivo*. The liver, lung, heart, spleen, and kidney were isolated from young mice (2 months old) and aged mice (24 months old) for immunohistochemistry (IHC) analysis (Fig. 6A–H). The results demonstrated a significant reduction in DHX9 expression in liver and lung (Fig. 6B–D), confirming our previous observations in BJ cells. However, DHX9 did not exhibit any noticeable changes in tissues with inherently low DHX9 expression (Fig. 6E–H). Meanwhile, we also assessed the rG4 levels in the tissues and observed a significant increase specifically in the liver and lung of aged mice (Fig. S8). Additionally, we isolated tail-tip fibroblast cells to investigate the level of DHX9, the abundance of rG4, and ribosome pausing (Fig. 6I). The results revealed that tail-tip fibroblast cells from aged mice exhibit a noteworthy reduction in DHX9 (Fig. 6J and 6K) alongside a substantial elevation in rG4 levels (Fig. 6L and 6M). Furthermore, we observed the activation of eIF2α, which led to the inhibition of overall translation in fibroblasts derived from aged mice (Fig. 6N–P). This phenomenon was consistent with the findings in BJ cells. In addition to ribosome pausing, there was an increase in aggresome formation in the fibroblasts of aged mice (Fig. 6Q and 6R), suggesting a disturbance in proteostasis within these cells. Collectively, these observations provide compelling evidence supporting the involvement of DHX9 in the regulation of proteostasis through rG4, both in senescent cells and in aged mice.

## Discussion

### Ribosome pausing in senescent cells

Ribosome pausing has emerged as a significant focus of research in recent years, primarily attributed to aberrant mRNA, which encompasses RNA modifications and secondary structure (Yan and Zaher, 2019). Various stress conditions have been reported to result in ribosome pausing and collisions, which may lead to diverse cell fates, such as apoptosis and senescence (Snieckute et al., 2023; Stein et al., 2022; Wu et al., 2020). Currently, investigations into ribosome pausing and cellular senescence predominantly emphasize the role of RNA modifications. Previous studies have indicated that N^6^-methyladenosine (m^6^A) modifications on mRNA play important roles in translation and cellular senescence (Boulias and Greer, 2023; Chen et al., 2022; Wang et al., 2015). Recently, N^1^-methylpseudouridylation of mRNA has been reported to cause +1 ribosomal frameshifting and influence mRNA translation (Mulroney et al., 2024), suggesting that it may play a role in cellular senescence. In our study, we have identified a novel mechanism of ribosome pausing, wherein the G-quadruplex structure in the mRNA coding region inhibits ribosome elongation, resulting in ribosome stalling and collisions. Furthermore, we observed a significant increase in the abundance of rG4 in the senescent cells, and the stabilization of the rG4 structures by cPDS further exacerbates ribosome pausing and contributes to cellular senescence, as illustrated in Figs. 3 and 4. These findings elucidate the previously unexplored impact of mRNA secondary structures on translation and cellular senescence.

### Role of RNA G-quadruplex in mRNA translation

Previous study has demonstrated that rG4 structures located in 5′UTR of mRNA play a crucial role in regulating translation initiation (Murat et al., 2018). These 5′UTR rG4 structures can recruit the 80S ribosome, thereby inhibiting gene translation. DExH/D helicases, including DHX36, DHX9, and DDX3X, are capable of unwinding rG4 structures located on the 5′UTR or rG4 structures on the 5′UTR of ribosomal proteins, thus facilitating translation process ( Murat et al., 2018; Varshney et al., 2021). However, existing research primarily focuses on the effects of rG4 structures in the 5′UTR on translation, with limited investigation into the influence of rG4 structures present within the coding regions of mRNA on translation efficacy. Here, we propose that rG4 structures located in the CDS of mRNAs can impede ribosome movement, resulting in ribosome pausing and collision. Stabilization of the rG4 structures by cPDS further exacerbates ribosome pausing.

Furthermore, a significant increase in the abundance of rG4 was observed in senescent cells. Combining this finding with prior research, we postulate that the reduction of helicases during the cellular senescence process could be the underlying cause. To pinpoint the potential helicase, we integrated ribo-seq and RNA-seq data and identified *DHX9* as the promising target. Upon validation, it was confirmed that DHX9 is indeed downregulated in senescent cells and aged mice (Figs. 5 and 6). Moreover, deficiency of the RNA helicase DHX9 resulted in an increased presence of rG4 structures, thereby exacerbating ribosome pausing. Conversely, the overexpression of DHX9 alleviated ribosome pausing (Figs. 5 and S6). These findings provide evidence that the presence of rG4 structures within the CDS of mRNAs can induce ribosome pausing and collisions.

### RNA G-quadruplex-binding peaks enrich on ribosome genes

There are ~360,000 potential sites for generating G-quadruplex in the genome, but not all genes are capable of forming these structures (Huppert and Balasubramanian, 2005). To identify the genes whose translation is regulated by rG4, G4P-RIP-seq was conducted to pull down the mRNAs associated with rG4. Subsequent analysis using GO and KEGG revealed that rG4 is enriched on ribosome genes in senescent cells (Fig. 2H and 2I), which is consistent with previous research. This suggests that rG4 initially decreases the TE of ribosomal proteins, potentially impacting ribosome assembly. Furthermore, the occurrence of a cascade reaction results in an insufficient number of functional ribosomes available for the translation of other genes, leading to the collapse of proteostasis that accelerates cellular senescence process.

### DHX9 captures mRNA containing rG4 by forming stress granules

DHX9 is the member of the DEAH-box RNA helicase family, which catalyzes the ATP dependent unwinding of RNA or DNA secondary structure. DHX9 plays important roles in many cellular processes, such as DNA replication, cell cycle, and mRNA translation (Hartman et al., 2006; Lee et al., 2014; Zhou et al., 2003). Previous study reported that deficiency of DHX9 induces premature senescence dependent on p53 (Lee et al., 2014). In this study, we demonstrate a novel mechanism of DHX9 in cellular senescence through unwinding rG4 structures to alleviate ribosome pausing. Recent study has shown that DHX9 can generate stress granules to sequester RNA damaged by ultraviolet light, thereby protecting progeny cells (Zhou et al., 2024). Additionally, under conditions of starvation or oxidative stress, there is a significant increase in the abundance of rG4 within mRNA (Kharel et al., 2023). Furthermore, some studies have suggested that rG4 might regulate the formation of stress granules (Asamitsu et al., 2023; Danino et al., 2023). Building upon these findings and our own research, it is possible that when rG4 causes ribosomal elongation pausing, DHX9 is likely to recognize and encapsulate these mRNA molecules within stress granules. These stress granules not only provide a more effective solution to ribosomal pausing but also prevent additional ribosomes from binding to the mRNA, thus avoiding new translation challenges. This aspect warrants further exploration and investigation.

### DHX9 and RNA G-quadruplex server as the potential targets for delaying cellular senescence

**Figure 7.**
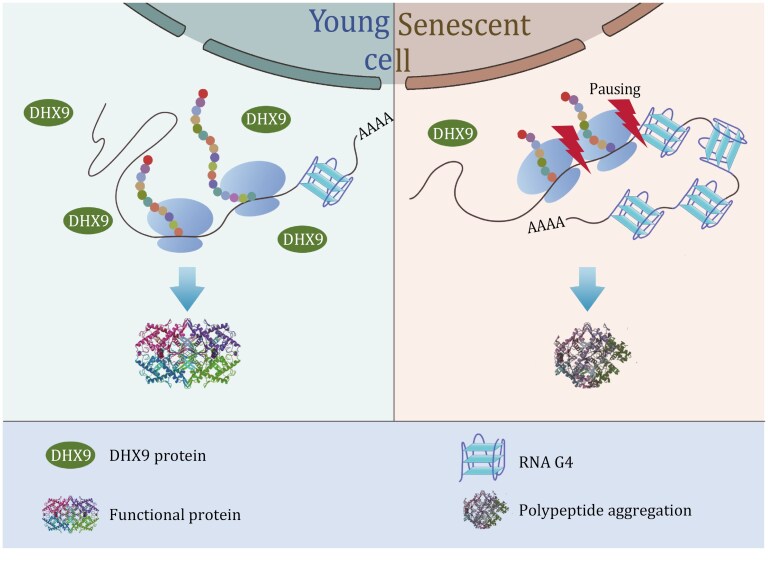
Proposed working model for RNA G-quadruplex to regulate cellular senescence. DHX9 functions to unwind mRNA rG4 structures, thereby preventing ribosome pausing during translation. In senescent cells, a decrease in the abundance of DHX9 protein results in the persistent presence of mRNA rG4 structures, leading to the halting of ribosomes. Consequently, the stalled ribosomes lead to collisions, ultimately contributing to the collapse of proteostasis. This figure was created with photoshop.

Cellular senescence constitutes a fundamental component of organismal aging, wherein the disruption of proteostasis emerges as a pivotal characteristic. Previous research has predominantly focused on factors such as UPR and autophagy (Pohl and Dikic, 2019; Senft and Ronai, 2015; Sha et al., 2017). Nevertheless, our study uniquely investigates the intricate process of protein synthesis, specifically in translation. Our findings reveal that the deficiency of DHX9 and the stabilization of rG4 both contribute to ribosome pausing, ultimately leading to proteostasis collapse. We propose that in young cells, an adequate presence of DHX9 facilitates the unwinding of rG4 in the mRNA, enabling ribosomes to glide smoothly and efficiently translate proteins. In contrast, senescent cells with a significant reduction of DHX9 exhibit insufficient capacity to unravel the elevated rG4, thereby resulting in ribosome pausing and abnormal protein translation (Fig. 7). Consequently, compounds that activate DHX9 or unwind rG4 hold promise for preserving proteostasis and delaying cellular senescence. Currently, G4 and rG4-related compounds are predominantly employed as stabilizing agents. Nonetheless, there is significant potential in exploring compounds that can unwind rG4, as they may serve as effective therapeutics for delaying cellular senescence.

## Supplementary data

Supplementary data is available at *Protein & Cell* online https://doi.org/10.1093/procel/pwaf047.

pwaf047_Supplementary_Materials

## Data Availability

The raw Ribo-seq, G4P-RIP-seq, and RNA-seq data have been submitted to NCBI GSE database. All relevant data are within the paper and its Supporting Information files. All packages used for data analysis are publicly available. Any additional information required to reanalyze the data reported in this paper is available from the lead contact upon request.

## Code availability

No customized code was generated for the present study.
