# Supplementary material for: RNA G-quadruplex (rG4) exacerbates cellular senescence by mediating ribosome pausing
Source: Protein Cell. 2025 Jun 12;16(11):953–67. doi: 10.1093/procel/pwaf047 (PMC12698187; doi:10.1093/procel/pwaf047)

Figure 3F

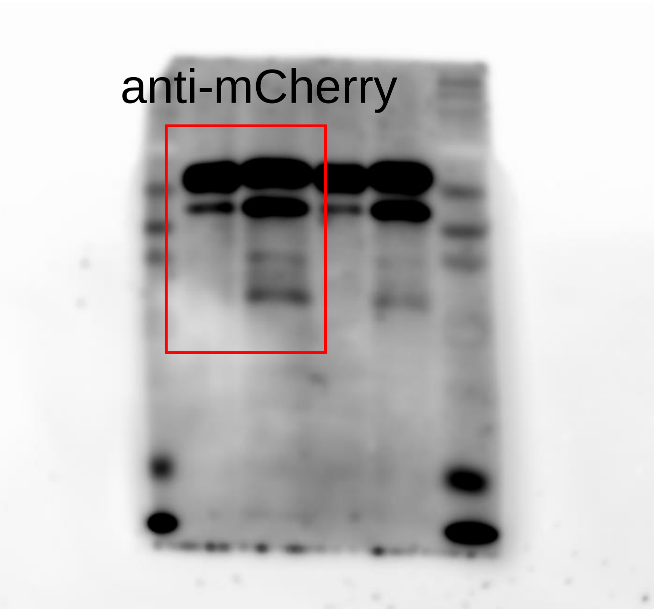

Figure 3H

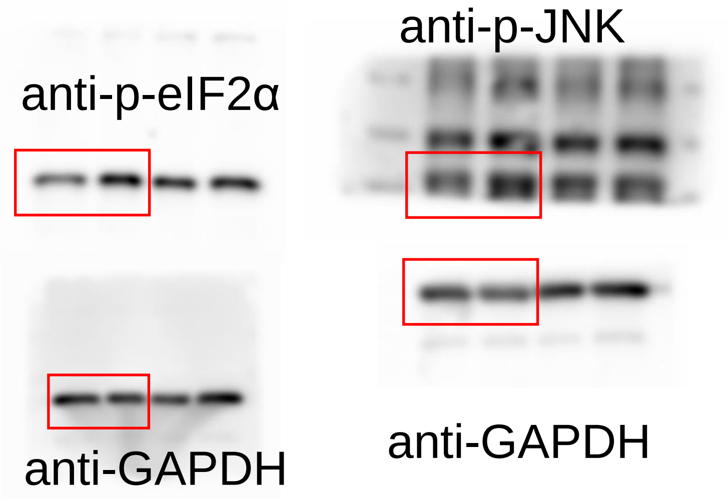

Figure 3G

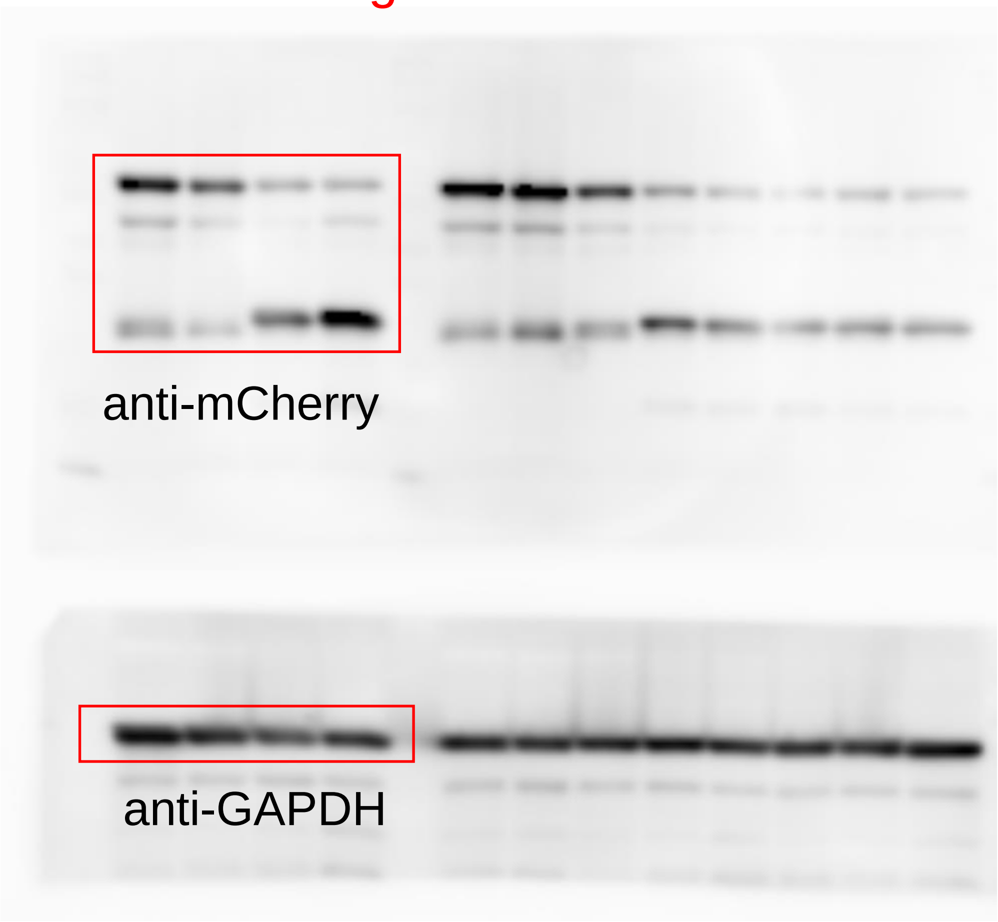

Figure 4F

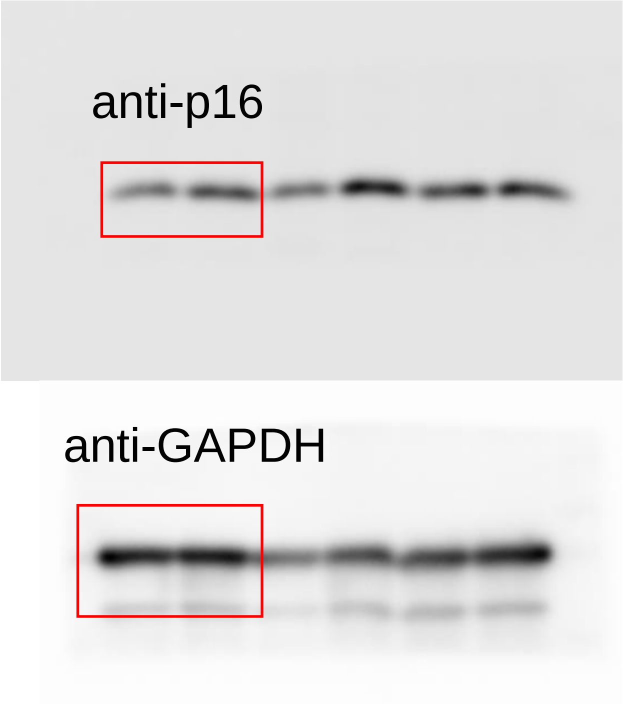

Figure 5D

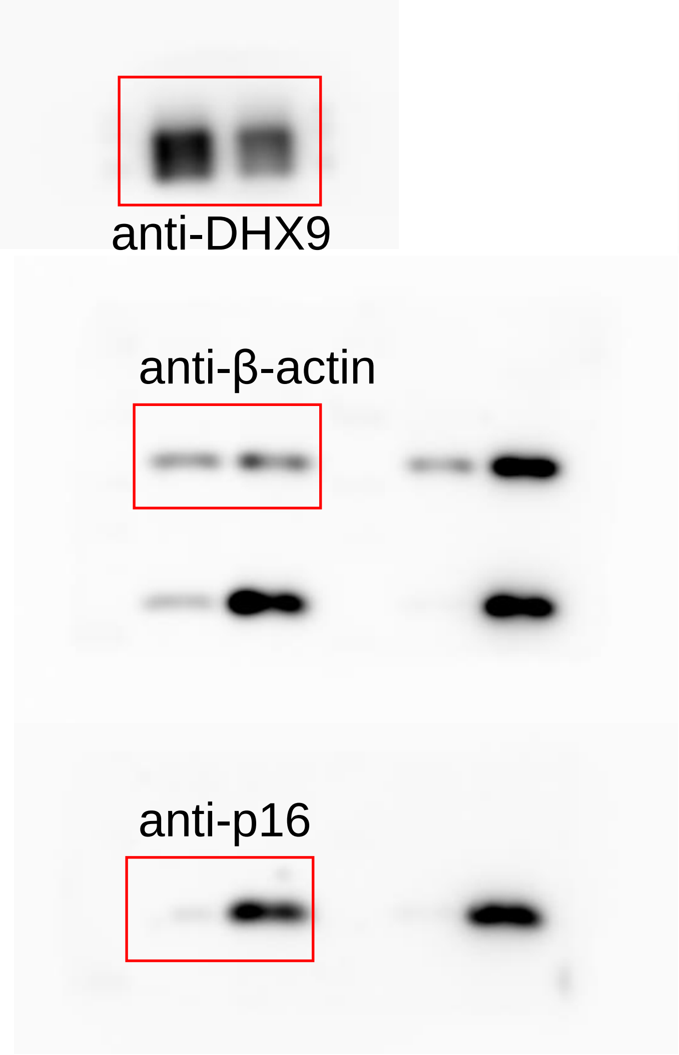

Figure 5I

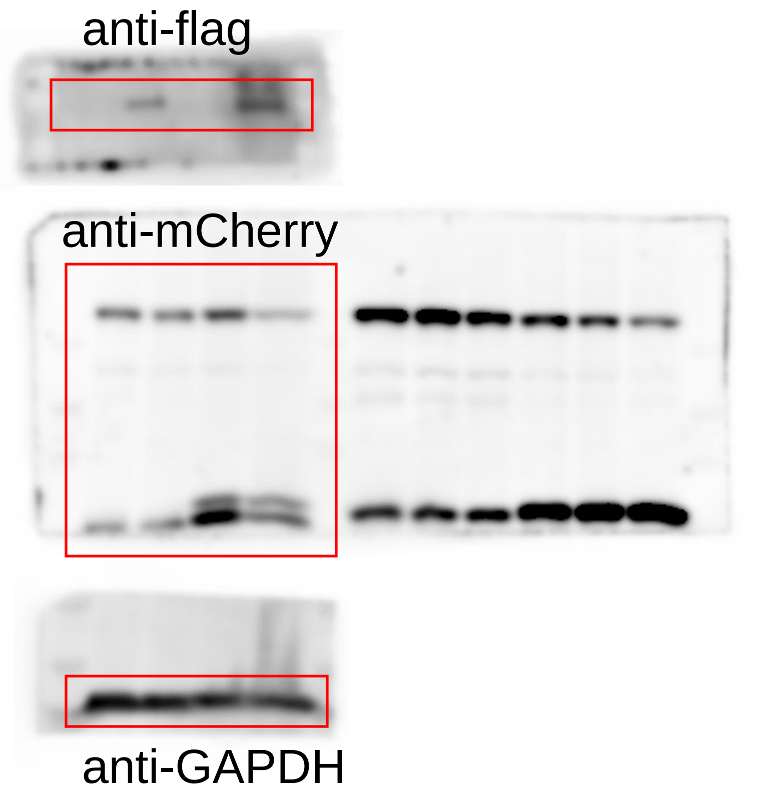

Figure 6J

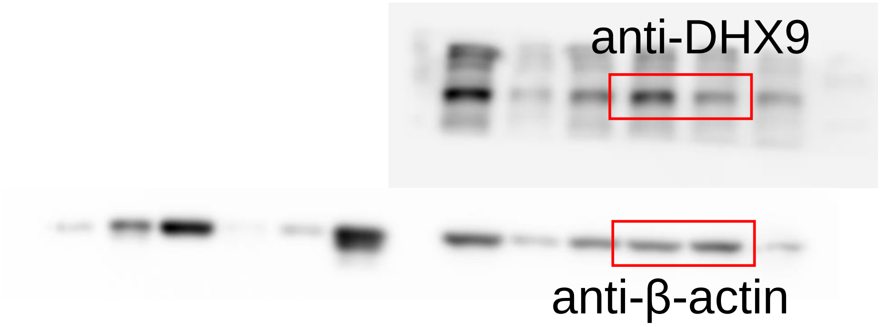

Figure 6N

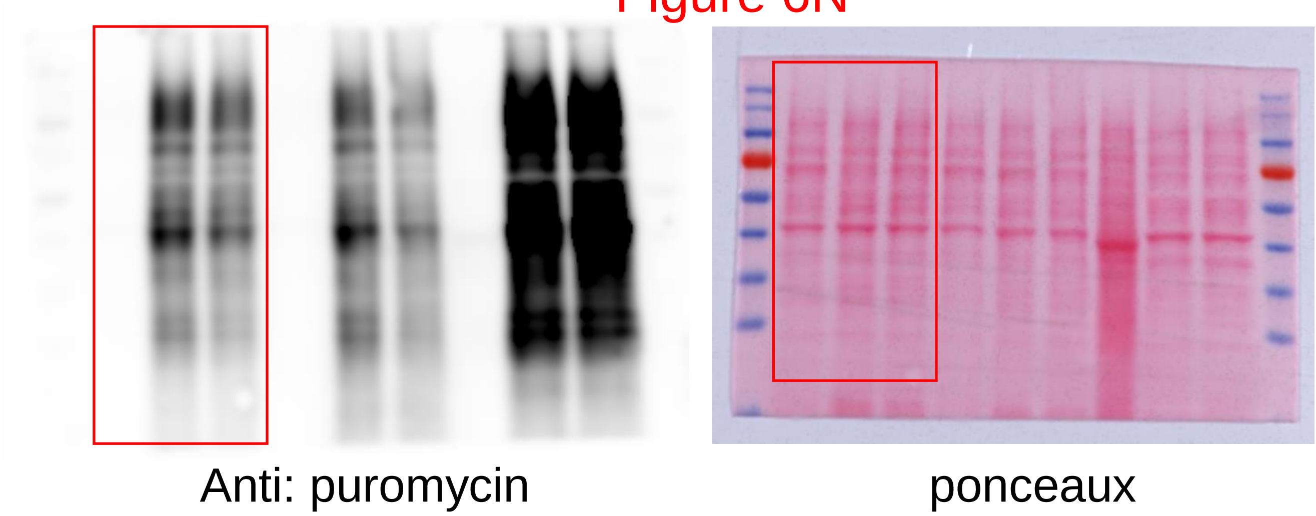

Figure 6O

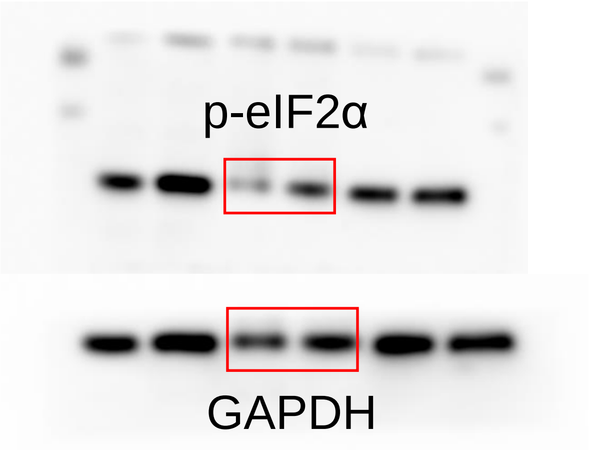

Figure S5A

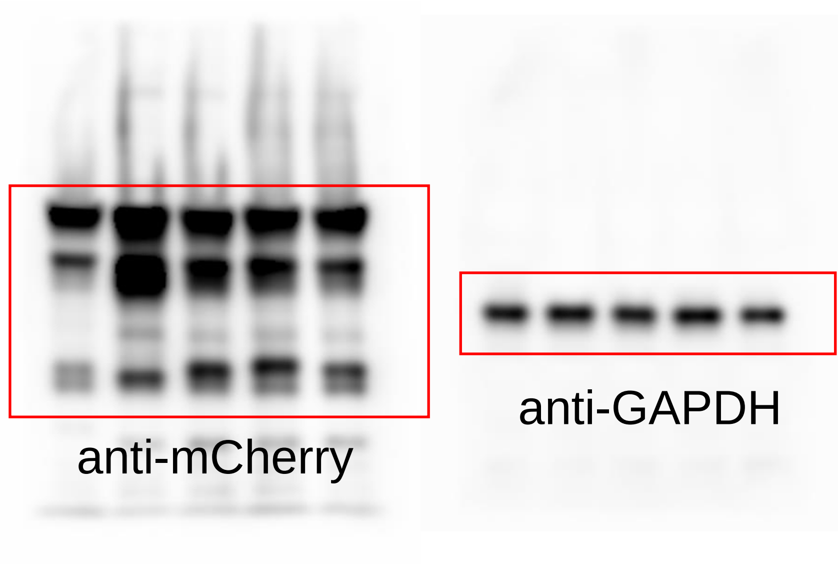

Figure S5C

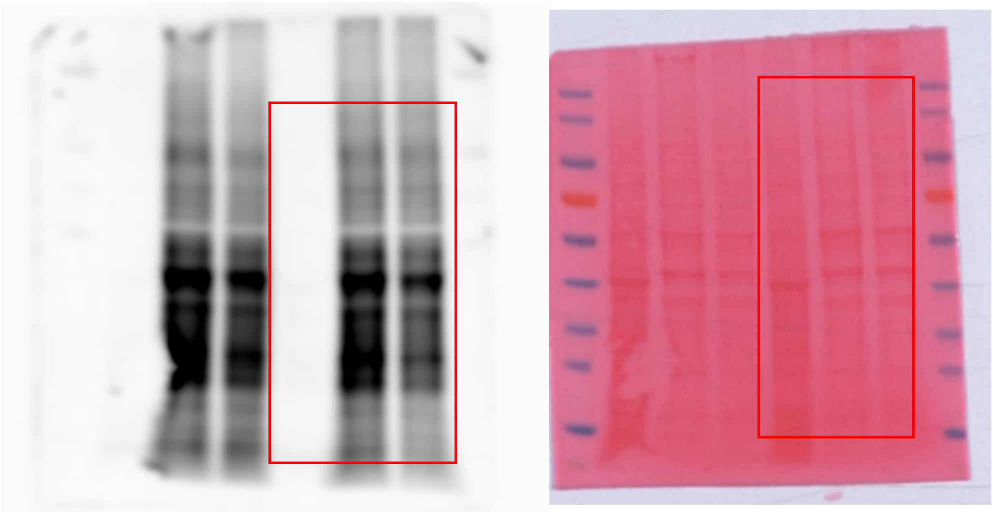

Anti: puromycin

ponceaux

Figure S7C

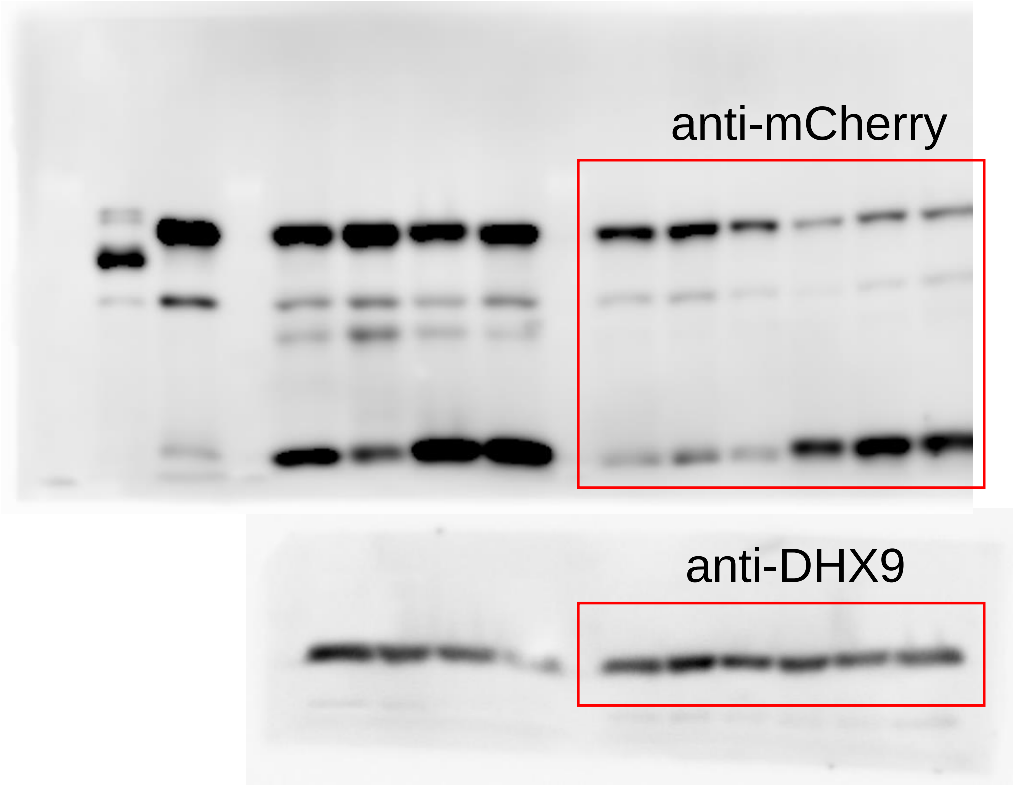

Figure S7D

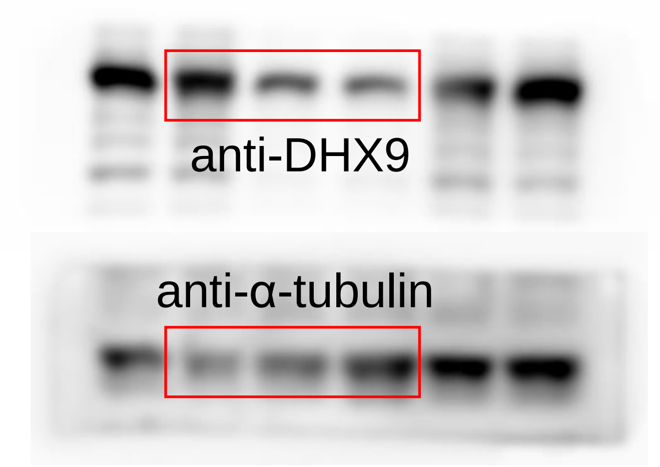

Supplement: pwaf047_Supplementary_Materials [file pwaf047_supplementary_materials.zip › WB source data.pdf]
