# Supplementary material for: RNA G-quadruplex (rG4) exacerbates cellular senescence by mediating ribosome pausing
Source: Protein Cell. 2025 Jun 12;16(11):953–67. doi: 10.1093/procel/pwaf047 (PMC12698187; doi:10.1093/procel/pwaf047)
Supplement: pwaf047_Supplementary_Materials [file pwaf047_supplementary_materials.zip › PAC-25078-JZY-Supplementary infomation.pdf]

SUPPLEMENTARY INFORMATION

RNA G-quadruplex (rG4) Exacerbates Cellular Senescence by Mediating Ribosome Pausing

Haoxian Zhou[1,2#], Shu Wu[2#], Bin Li[3#], Rongjinlei Zhang[2], Ying Zou[2], Mibu Cao[7] , Anhua Xu[2], Kewei Zheng[4], Qinghua Zhou[5], Jia Wang[6], Jinping Zheng[6], Jianhua Yang[3], Yuanlong Ge[2]*, Zhanyi Lin[1]*, Zhenyu Ju[2]*

[1]Department of Cardiology, Guangdong Provincial Cardiovascular Institute, Guangdong Provincial People's Hospital, Guangdong Academy of Medical Sciences, Guangzhou, China;

[2]Key Laboratory of Regenerative Medicine of Ministry of Education, Institute of Aging and Regenerative Medicine, Department of Developmental & Regenerative Medicine, College of Life Science and Technology, Jinan University, Guangzhou, China;

[3]School of Life Sciences, Sun Yat-sen University, Guangzhou, China;

[4]School of biomedical sciences, Hunan University, Changsha, China;

[5]Key Laboratory of Regenerative Medicine of Ministry of Education, Institute of Aging and Regenerative Medicine and The First Affiliated Hospital, Jinan University, Guangzhou 510632, Guangdong, P.R.China;

[6]Shanxi Key Laboratory of Aging Mechanism Research and Translational Applications, Changzhi Medical College, Changzhi, Shanxi, China;

[7]Department of Plastic and Reconstructive Surgery, Guangdong Second Provincial General Hospital, 510317, Guangzhou, China.

*To whom correspondence should be addressed, Email: zhenyuju2016@jnu.edu.cn
Correspondence may also be addressed to Zhanyi Lin. Email: linzhanyi@gdph.org.cn
Yuanlong Ge. Email: geyuanlong@jnu.edu.cn
# These authors contributed equally to this work.

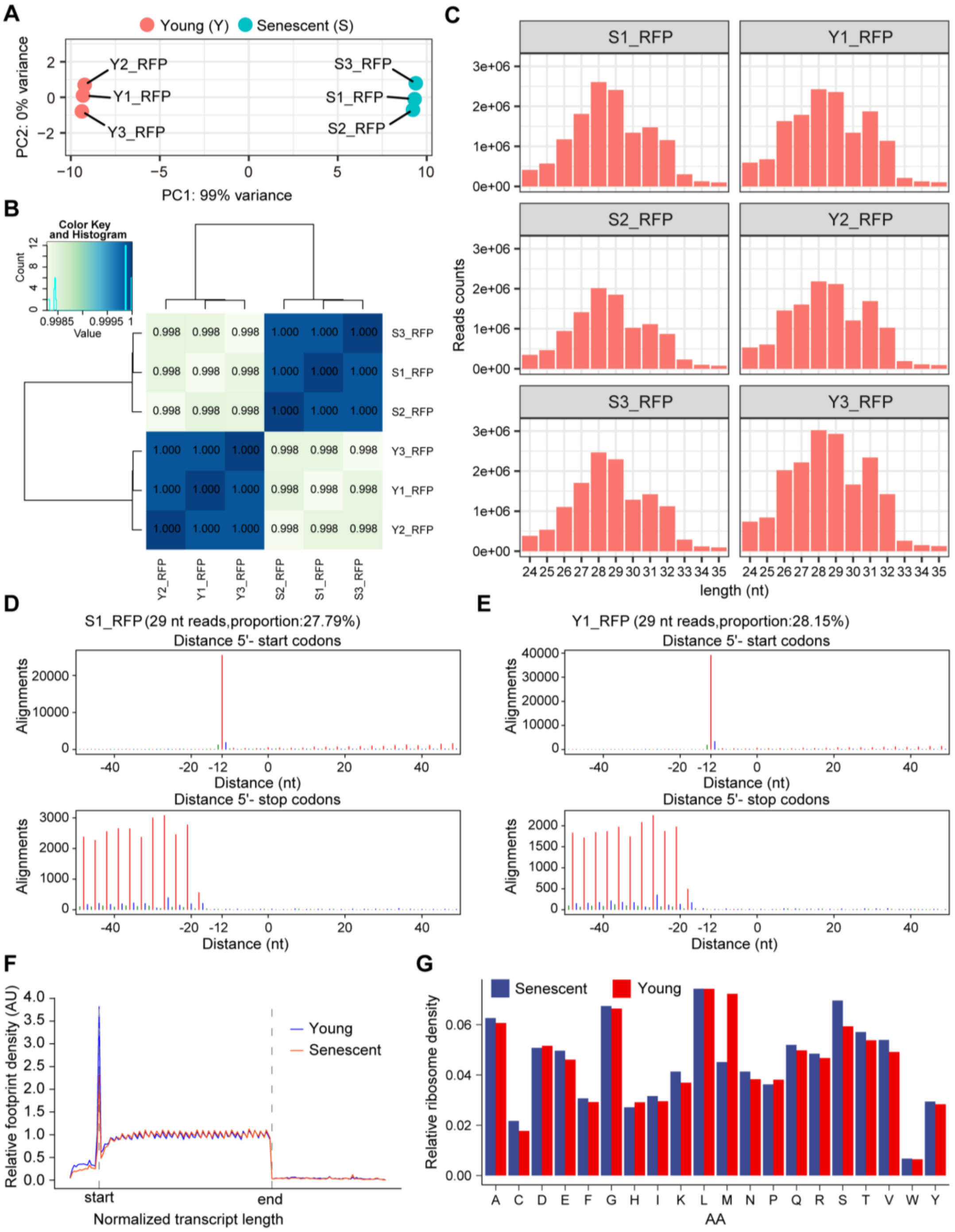

Fig. S1 Genome-wide transcriptome analysis of young and senescent cells.

(**A**) Principal Component Analysis (PCA) displaying the variance within and between the senescent and young BJ cell Ribo-seq samples. (**B**) Sample correlation analysis of Ribo-seq replicates from senescent and young BJ cells. (**C**) Length distribution of RPFs in young and senescent BJ cells. (**D-E**) Analysis of 3-nt periodicity patterns in 29-nt reads from senescent and young BJ cell Ribo-seq (replicate 1) using RiboCode. (**F**) Overall read density distributions along all the transcripts merged during BJ cell senescence. (**G**) Relative ribosome density on each amino acid for senescent and young BJ cells.

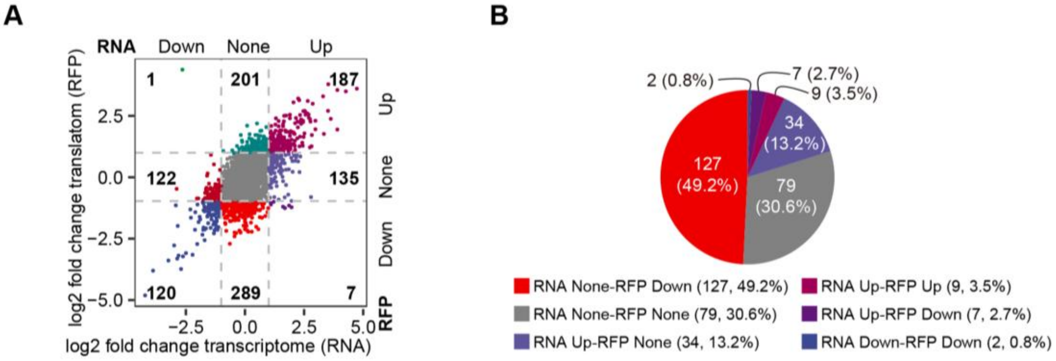

C

Motifs	N	M	n	m	p value
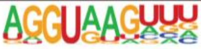	9158	7	3508	122	< 2.22e-16
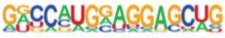	9158	6	3508	13	< 2.22e-16
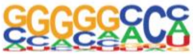	9158	286	3508	128	1.38e-2

Fig. S2 Analysis of transcriptome and translatome change patterns in genes exhibiting reduced translational efficiency.

(**A**) Scatterplot illustrating log2-transformed fold changes in the transcriptome and translatome. This scatterplot represents the alterations in ribosome footprint counts (translatome) plotted against RNA abundance (transcriptome) in senescent BJ cells when compared to their younger counterparts. Genes displaying substantial changes in either RFP or RNA abundance (⩾2-fold changes) are visually emphasized with color, while genes exhibiting minimal changes are labeled in gray. (**B**) Pie plot depicting the changes in translation (RFP) and transcription (RNA) of genes with downregulated translation efficiency. **(C)** The enrichment significance of motif sequences in the 20-nt sequences downstream of ribosome stalling sites was assessed using Homer. In this context, N represents the 20-nt sequences randomly selected from the CDS of all translatable genes in BJ cells (excluding the 50-nt sequences downstream of the start codon and upstream of the stop codon). M denotes the number of sequences in N that match the motif as identified by Homer. n represents the number of 20-nt sequences downstream of ribosome stalling sites that are significantly upregulated in senescent cells compared to young cells. m denotes the number of sequences in n that match the motif as identified by Homer. The p-value is calculated using the hypergeometric distribution test.

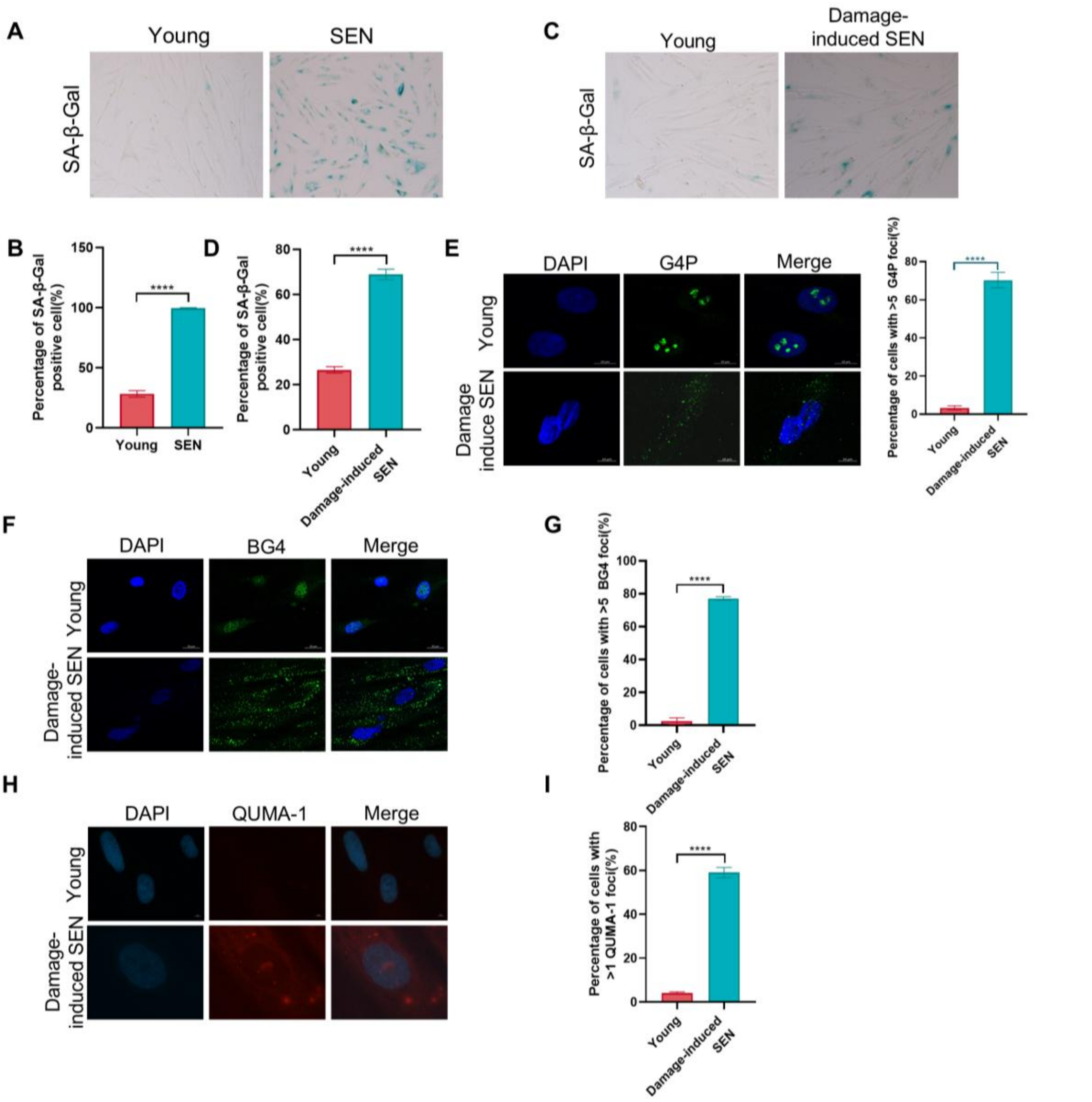

Fig. S3 RNA G-quadruplex is increased in damage-induced senescent cells.

(**A**) SA-β-Gal staining of replication senescent cells. (**B**) Quantification of (A). SA-β-Gal positive cells were calculated (n ≥ 100 cells). All values are means ± SEM of more than three independent experiments. (**C**) SA-β-Gal staining of damage-induced senescent cells. BJ cells were treated with VP-16 (40 μM) for 24 h and then released for 48 h to induce cellular senescence. (**D**) Quantification of (C). SA-β-Gal positive cells were calculated (n ≥ 100 cells). All values are means ± SEM of more than three independent experiments. (**E**) Cytoplasm G4P signal was increased in damage-induced senescent cells. BJ cells were treated with VP-16 (40 μM) for 24 h and then released for 48 h to induce cellular senescence. G4P was induced by DOX and IF was performed with Flag antibody. Cells contain more than one cytoplasm G4P foci were calculated (n ≥ 100 cells). All values are means ± SEM of more than three independent experiments. (**F**) Cytoplasm BG4 signal was increased in damage-induced senescent cells. IF was performed with BG4 antibody. (**G**) Quantification of (F). Cells contain more than one cytoplasm BG4 foci were calculated (n ≥ 100 cells). All values are means ± SEM of more than three independent experiments. (**H**) rG4 was increased in damage-induced senescent cells. Imaging of damage-induced senescent cells stained with QUMA-1(2 μM) for 20 mins at 37°C. (**I**) Quantification of (H). Cells contain more than one cytoplasm QUMA-1 foci were calculated (n ≥ 100 cells). All values are means ± SEM of more than three independent experiments.

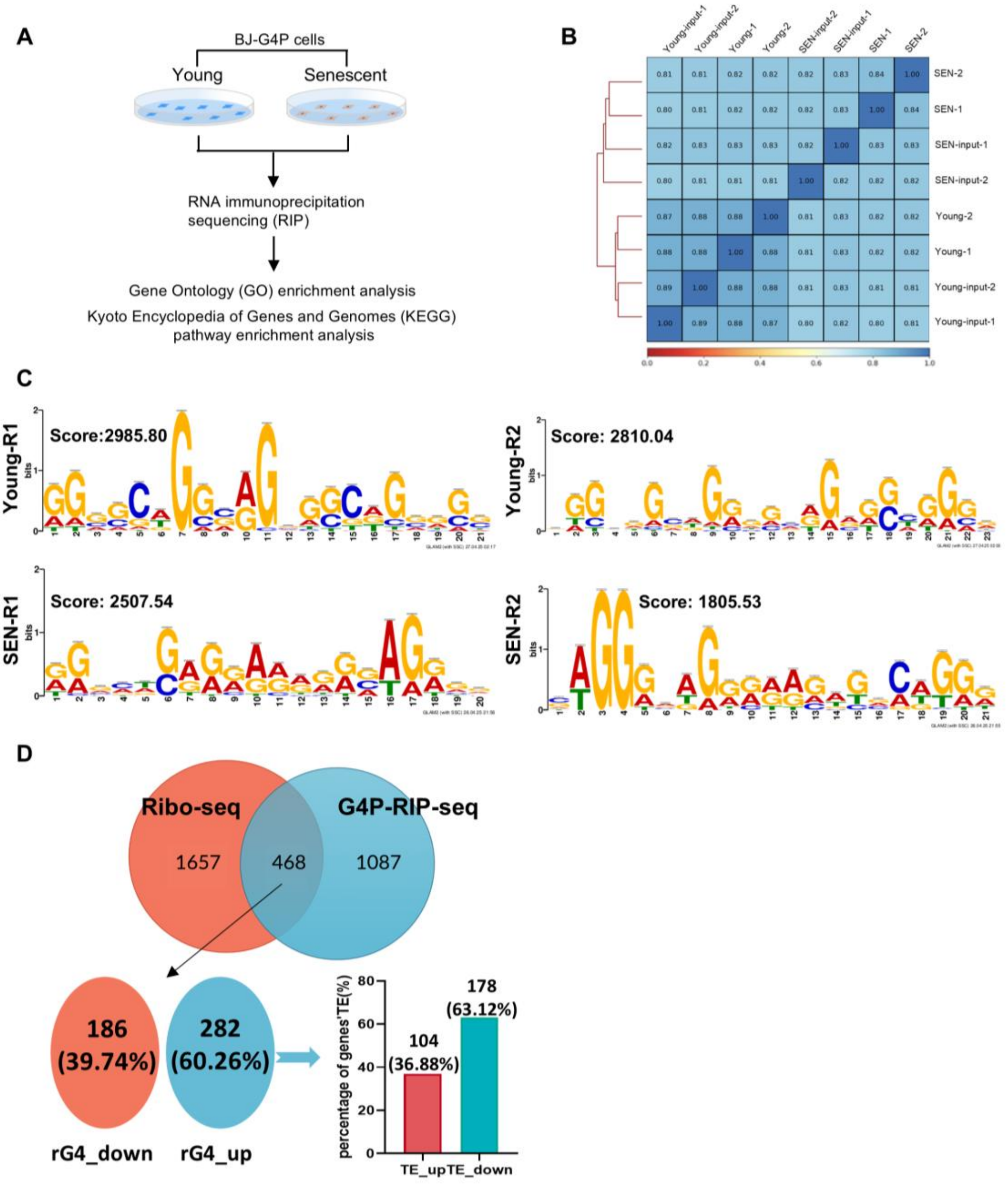

Fig. S4 The quality of G4P RIP-seq in young and senescent cells.

(**A**) Schema illustrating the workflow for G4P-RIP-seq in young and senescent cells. (**B**) Sample correlation analysis. (C) The peak sequences obtained from G4P-RIP-seq data were used to analyze motifs using GLAM2. (**D**) Most intersection genes between Ribo-seq and G4P-RIP-seq have increased rG4 level and decreased translation efficiency.

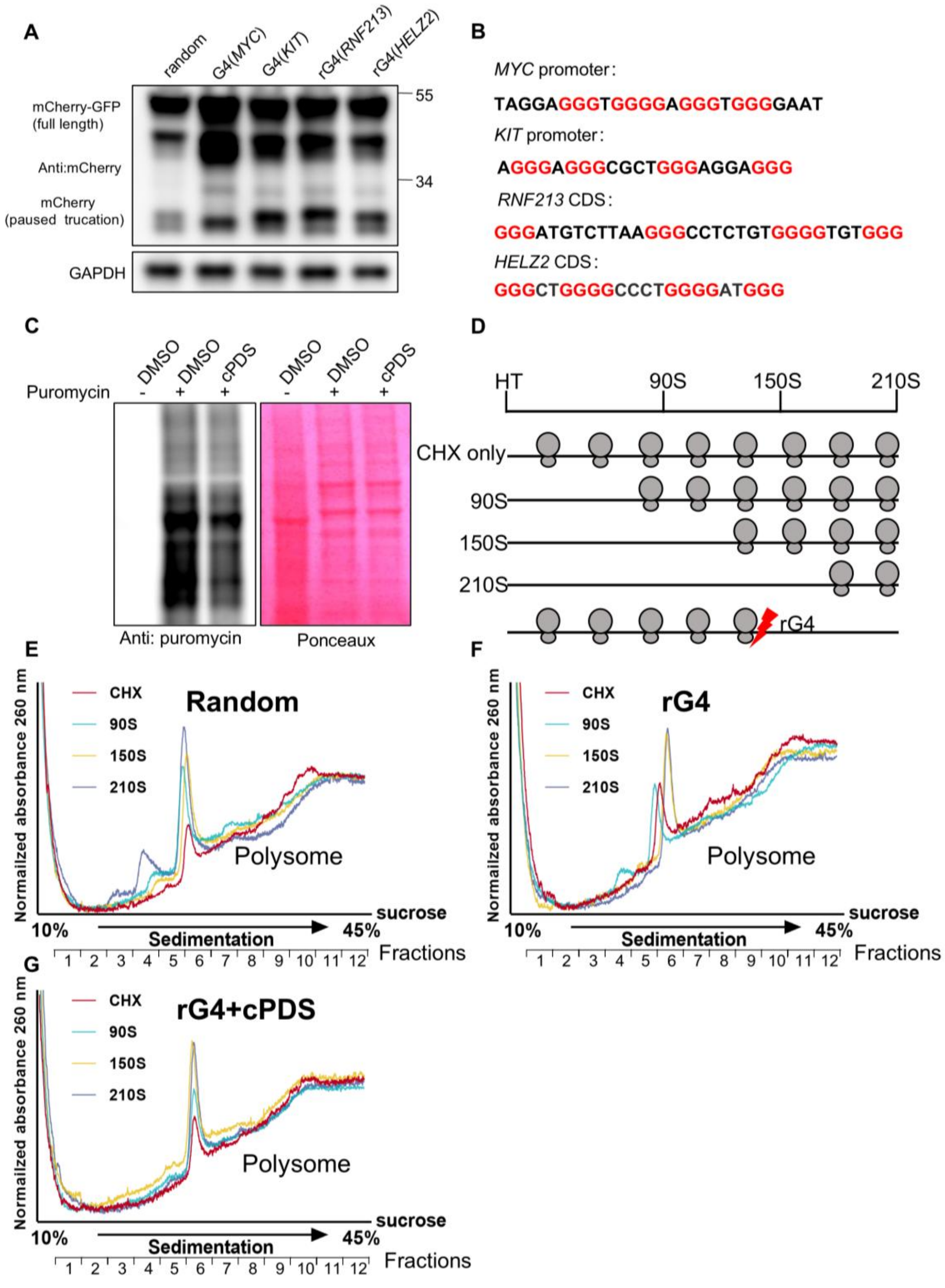

Fig. S5 RNA G-quadruplexes exacerbate ribosome pausing leading to translation impairment.

(**A**) Immunoblot of dual fluorescence reporter containing the indicated rG4 sequences in 293T cells. 293T cells were transfected with the dual fluorescence reporter for 72 hours. Western blot analysis was performed with the indicated antibodies, with truncated and full-length products labeled. (**B**) Four bona fide rG4 sequences incorporated into the dual fluorescence reporter. (**C**) Puromycin incorporation assay in pre-senescent BJ cells showed reduced Puromycin incorporation after cPDS treatment. Western blot analysis was performed with the indicated antibodies, with the right panel

displaying the Ponceau S-stained membrane for the same blot. (D) Schematic diagram of the ribosome run-off experiment design. (E-G) 293T cells were transfected with dual fluorescence reporter system and treated with or without cPDS. 293T cells were treated with harringtonine(2μg/mL) and subsequently collected for ribosome profiling according to schematic A. Shown is the representative UV trace of polyribosome gradients from 293T cell lysates of each group.

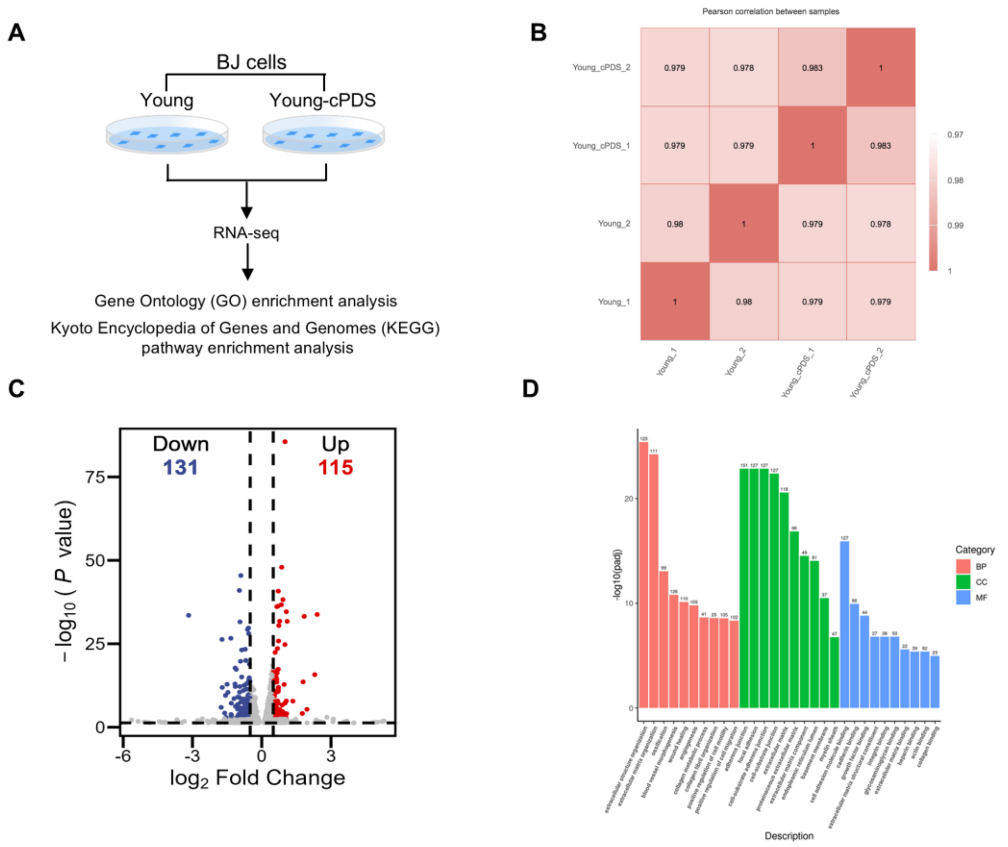

Fig. S6 The quality of RNA-seq in young and cPDS-treated cells.

(**A**) Schema illustrating the workflow for RNA-seq in DMSO and cPDS-treated young BJ cells. (**B**) Sample correlation analysis. (**C**) Volcano plot illustrating changes in transcription level between DMSO and cPDS treatment BJ cells (with cutoffs set at $p < 0.05$ and |log2TE-fold change| ≥0.5). (**D**) GO functional enrichment analysis of differentially enrich genes between DMSO and cPDS-treated young BJ cells.

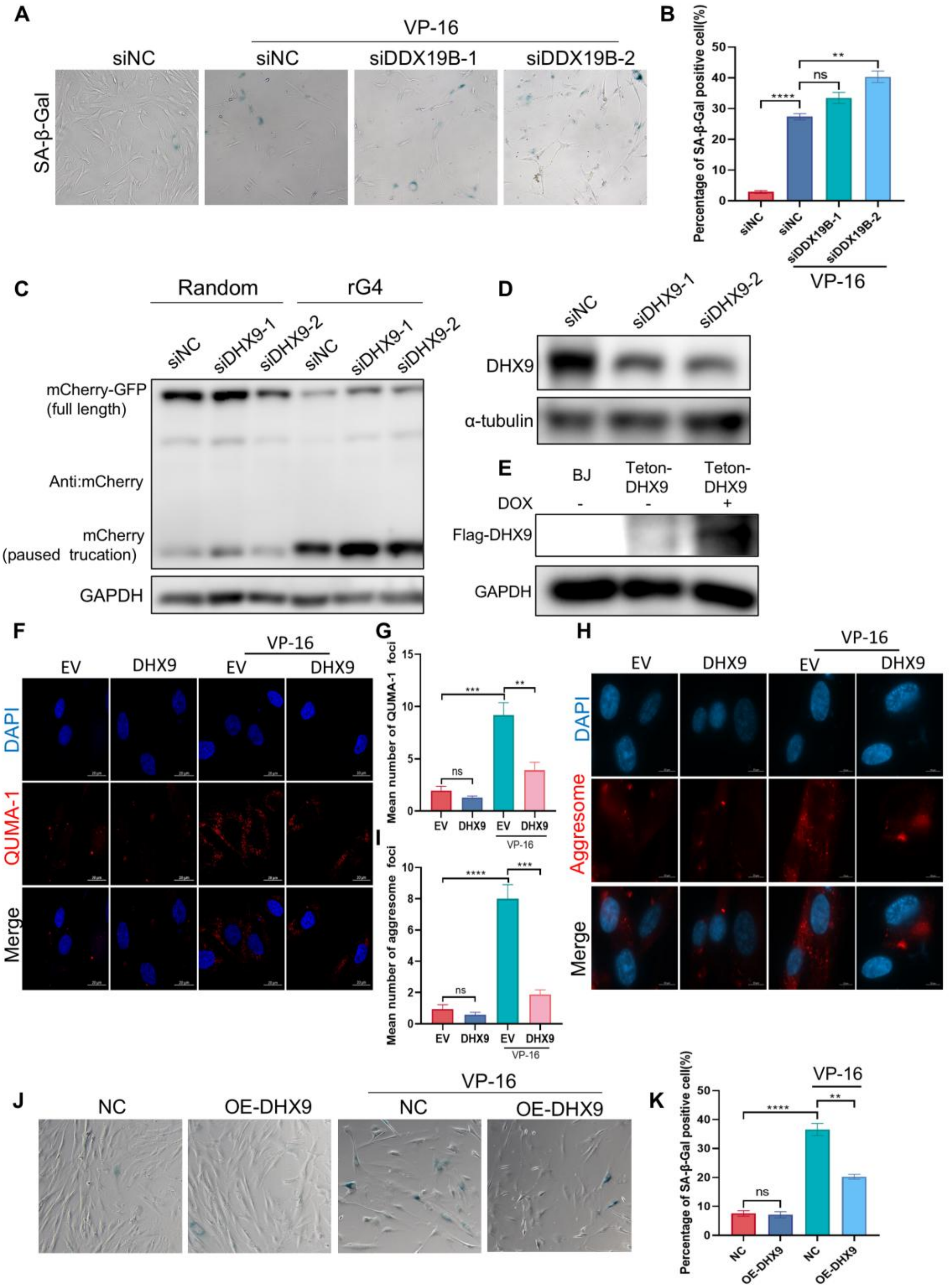

Fig. S7 DHX9 deficiency facilitates RNA G-quadruplex stability and cellular senescence.

(**A**) DDX19B deficiency slightly accelerates cellular senescence. BJ cells were treated with VP-16 (40 μM, 24 h) and transfected with siRNAs (72 h), then stained by SA-β-Gal. (**B**) Quantification of (A). SA-β-Gal positive cells were calculated (n≥100 cells). All values are means ± SEM of more than three independent experiments. (**C**) Immunoblot of dual fluorescence reporter in DHX9 deficiency 293T cells. 293T cells were transfected with dual fluorescence reporter and siRNAs for 72 h. Western was performed with indicated antibodies. Truncated and full-length products are noted.

n=3 biological replicates with representative example shown. (**D**) Immunoblot analysis of indicated proteins in DHX9 deficiency BJ cells. BJ cells were transfected with siRNAs for 72 h (n=3). (**E**) Immunoblot analysis of indicated proteins. 293T cells were transfected with Flag-DHX9 plasmid for 72 h. (F) Imaging of replication senescent cells stained with QUMA-1 (2 μM) for 20 mins at 37°C. (G) Quantification of (F). Cells contain more than one cytoplasm QUMA-1 foci were calculated (n⩾100 cells). All values are means ± SEM of more than three independent experiments. (H) Representative aggresome staining images of DHX9-overexpression cells. (I) Quantification of (H). Aggresome decreased in DHX9-overexpression cells. Cells contain more than one aggresome foci were calculated (n⩾100 cells). All values are means ± SEM of more than three independent experiments. (**J**) Overexpression of DHX9 delay cell senescence. BJ cells were treated with VP-16(40 μM, 24 h) and overexpressed with DHX9(72 h), then stained by SA-β-Gal. (**K**) Quantification of(J). SA-β-Gal positive cells were calculated (n ≥ 100 cells). All values are means ± SEM of more than three independent experiments.

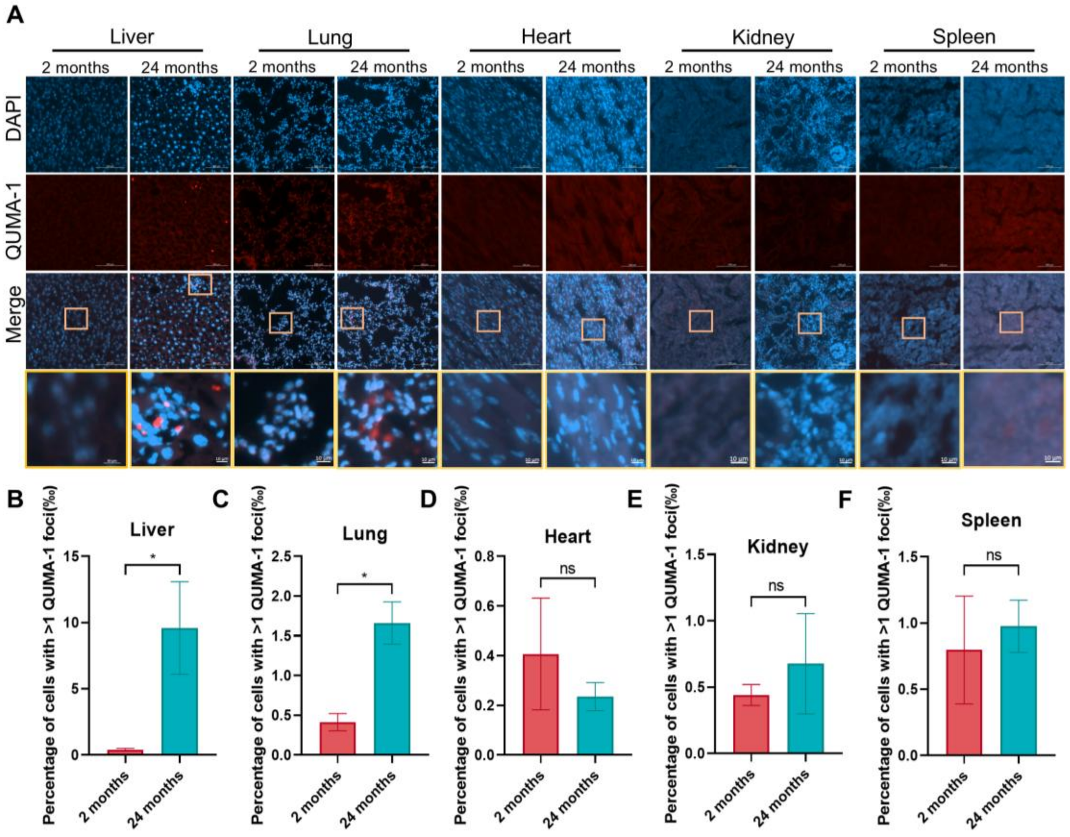

Fig. S8 RNA G-quadruplex increases in the liver and lung of aged mice.

(**A**) Immunofluorescence staining of QUMA-1 in the liver, lung, heart, spleen and kidney. (**B-F**) Quantification of (A). The number of cells contain more than one QUMA-1 foci were calculated. All values are means ± SEM of more than three mice.

RNA G-quadruplex (rG4) Exacerbates Cellular Senescence by Mediating Ribosome Pausing

Haoxian Zhou[1,2#], Shu Wu[2#], Bin Li[3#], Rongjinlei Zhang[2], Ying Zou[2], Mibu Cao[7] , Anhua Xu[2], Kewei Zheng[4], Qinghua Zhou[5], Jia Wang[6], Jinping Zheng[6], Jianhua Yang[3], Yuanlong Ge[2]*, Zhanyi Lin[1]*, Zhenyu Ju[2]*

[1]Department of Cardiology, Guangdong Provincial Cardiovascular Institute, Guangdong Provincial People's Hospital, Guangdong Academy of Medical Sciences, Guangzhou, China;
[2]Key Laboratory of Regenerative Medicine of Ministry of Education, Institute of Aging and Regenerative Medicine, Department of Developmental & Regenerative Medicine, College of Life Science and Technology, Jinan University, Guangzhou, China;
[3]School of Life Sciences, Sun Yat-sen University, Guangzhou, China;
[4]School of biomedical sciences, Hunan University, Changsha, China;
[5]Key Laboratory of Regenerative Medicine of Ministry of Education, Institute of Aging and Regenerative Medicine and The First Affiliated Hospital, Jinan University, Guangzhou 510632, Guangdong, P.R.China;
[6]Shanxi Key Laboratory of Aging Mechanism Research and Translational Applications, Changzhi Medical College, Changzhi, Shanxi, China;
[7]Department of Plastic and Reconstructive Surgery, Guangdong Second Provincial General Hospital, 510317, Guangzhou, China.

Materials and Methods

Ethics Statement

C57 BL/6J mice aged between 2-24 months were obtained from GemPharmatech (Guangdong, China). Mice were bred and housed at 25 °C, 12:12 h light:dark cycle under specific pathogen-free conditions at Jinan University laboratory animal center. All experiments were approved by the Animal Protection and Ethics Committee of Jinan University (102305, IACUC-202300509-09).

Cell culture

BJ fibroblast, 293T were obtained from Chinese Academy of Sciences of Type Culture Collection and were cultured at 37˚C under 5% CO2. Cells were cultured in DMEM (Gibco) with 10% fetal bovine serum (ExCell), 100 U/ml penicillin and 1% streptomycin (Gibco).

RNA-seq

Three micrograms of total RNA were utilized for polyadenylated RNA enrichment with VAHTS mRNA Capture Beads (Vayzme) following the manufacturer's protocol. The NEBNext Ultra II Directional RNA Library Prep Kit standard protocol (NEB) was employed for library preparation. Subsequently, the libraries were subjected to sequencing on an Illumina HiSeq X Ten platform, generating 150-bp paired-end reads at Annoroad Gene Technology Company.

Ribosome profling

Ribosome profiling (Ribo-seq) was performed as previous protocol(Xu et al., 2022, Yan et al., 2021) with minor modifications. Briefly, cell samples were treated with 100 µg/ml cycloheximide for 2 minutes at 37°C. CHX-treated cells were lysed using ice-cold mammalian polysome buffer (comprising 20 mM Tris-HCl, pH 7.5, 150 mM NaCl, 5 mM MgCl2, with 1 mM DTT, 1% Triton X-100, 0.1% IGEPAL, 500 U/mL RNase Inhibitor, 1× EDTA-free Complete protease inhibitors, 1 U/µl DNase I, and 100 µg/ml cycloheximide). After cell dispersion, the lysate was passed through a 26-gauge needle ten times and then clarified by centrifugation at 20,000g for 5 minutes. Approximately 120 µL of the supernatant was treated with 3 µL of RNase I (Life Technologies, AM2294) for 45 minutes at room temperature. To halt the reaction, SUPERase•In RNase Inhibitor (Life Technologies, AM2694) was added. The resulting monosomes were purified using MicroSpin S-400 HR columns (GE Healthcare, 27-5140-01). RNA was isolated from the flow-through (approximately 125 µL) using 1 mL of RNAzol. For enrichment of ribosome footprints (RFPs), RNA samples underwent treatment with the biotin-labeled probes specific for a variety of rRNA sequences to eliminate ribosomal RNA fragments. The remaining RNA was separated on a 17% 7.5M Urea-PAGE gel. A size range of 26 nt to 30 nt, defined by loading with the 14-30 ssRNA Ladder Marker (Takara, #3416), was excised, and ribosome profiling libraries were prepared as previously described. These libraries were subsequently sequenced on the HiSeq X Ten platform by Annoroad.

Ribosome run-off

The dual fluorescence reporter system was transfected into 293T cells. Prior to collection, cells were treated with cPDS (2 µM) for 24 hours. At 48 hours post-transfection, the culture medium was supplemented with 2 µg/mL harringtonine and

returned to 37°C. After the designated exposure time (90 s, 150 s and 210 s), 100 μg/mL cycloheximide was added to the medium to halt further translation elongation, followed by incubation at 37°C for 10 minutes. Cells were then collected for ribosome profiling, as described above.

RNA-Seq data analysis

Demultiplexed sequencing reads were processed by trimming adapter sequences using Cutadapt (v2.0.dev0)(Martin, 2011). Reads that aligned to the human genome (hg38) were identified using STAR (v2.4.2a)(Dobin et al., 2013) with ENCODE standard options for the long RNA-seq pipeline, employing the GENCODE transcriptome annotation (GENCODE v32) as a reference. Read counts were generated through featureCounts (v1.6.4)(Liao et al., 2014) with default settings. Significantly differentially expressed (DE) genes were analyzed using DESeq2(Love et al., 2014) and selected based on a fold change greater than 2 and an adjusted p-value less than 0.05.

Ribo-seq analysis and RPScore calculation

For the Ribo-seq data, Cutadapt (v2.0.dev0) was employed to trim relevant adapter sequences, eliminate low-quality reads, and filter out reads falling outside the 24-35 nt range. Reads aligning to rRNA were removed using Bowtie (v1.1.2)(Langmead et al., 2009) with default parameters. The remaining reads were subsequently mapped to the human genome (hg38) using STAR (v2.4.2a) with default options, except for the following settings: --outFilterMismatchNmax 2, --outFilterMultimapNmax 5, --outFilterMatchNmin 16, --alignEndsType EndToEnd. ORFs were identified and quantified following the guidelines provided in the RiboCode(Xiao et al., 2018) manual (version 1.2.15) with the following parameters: -l no -m 10 -A CTG,GTG,TTG -g -b. Translation efficiency (TE) is the ratio of RPF and RNA abundance determined by Ribo-seq and RNA-seq. Translation efficiency of ORFs was then calculated using Xtail(Xiao et al., 2016). Genes with differential translation efficiency (TE) were identified based on a fold change greater than 2 and an adjusted p-value less than 0.05. Similarly, when separately analyzing the differential changes in RNA and RFP levels, the screening threshold was also set at a fold change greater than 2 and a p-value less than 0.05. Ribosome density on each amino acid and across transcripts were analyzed

using RiboMiner(Li et al., 2020) (v 0.2.3.2). Ribosome pause scores (RPScores) at each site in a transcript were calculated by dividing the rpm (reads per million) associated with each nucleotide by the average rpm of the entire open reading frame, excluding the first 50 and last 50 sense nucleotides. RPScores at each codon position were the mean of the three nucleotides. To identify positions with increased ribosome pausing during cellular senescence, we initially considered genes with a reads per kilobase of exon per million reads mapped (RPKM) greater than 20. Subsequently, we applied the two-sided Wilcoxon test to pinpoint positions exhibiting statistically significant changes in ribosome pausing between young and senescent cells. Senescence-dependent pause sites met the following criteria: (1) p-value < 0.05; (2) a pause score in senescent samples at least twofold higher than in young samples; (3) pause scores with the same fold change retained the sites nearest to the stop codon. To identify potential motifs associated with ribosome pausing, we conducted motif enrichment analysis using the Homer software(Heinz et al., 2010) on the downstream sequences of ribosome pausing sites that were significantly enriched in senescence. The significance of the enrichment of these motifs was confirmed by the hypergeometric distribution test implemented in Homer.

Gene silencing and overexpression

Plasmid DNA was transiently transfected into 293T cells using the PEI (Yeasen) method: plasmid DNA was incubated with PEI for 15 min, added to cells at appropriate confluence (50–60%) and incubated for 8 h. The medium was exchanged for fresh medium, and cells were incubated for 48 h.

DHX9, DDX19B were deleted by siRNAs using Lipofectamine RNAiMAX Transfection Reagent (Invitrogen) according to the manufacturer's instructions.

siRNAs were provided by Suzhou GenePharma Co., Ltd.: DHX9-1:5′-UAGAAU GGGUGGAGAAGAA-3′; DHX9-2:5′-GGCUAUAUCCAUCGAAAUUUU-3′; DD X19B-1: 5′-CAUCCAAGAUACAAGAGAA-3′; DDX19B-2: 5′-CGGCAUUGAU GUUGAACAA-3′. The scrambled sequence was used as a control.

Dual fluoresence reporter

The dual fluoresence reporter was generated by insert the "mCherry-random" or "mCherry-rG4" sequence based on pLVX-AcGFP-N1 plasmid.

Random:
TATAGCGTCCTCGTAAAACGGAATCGGGTGTTGGACTATGCTTCTAA
G4:
GGGTAGGGTCGGGTAGGGTCCCATGGTGAGCAACGGGTAGGGTCGGGTA
GGGC

Cell-free protein synthesis

Cell-free protein synthesis was performed using the Premium ONE Expression Kit (TIANGEN BIOTECH). In brief, the WEPRO TTmix and SUB-AMIX TT were thawed on ice. Subsequently, 50 μl of SUB-AMIX TT was added to a 0.2 ml PCR tube. Separately, 2.5 μl of WEPRO TTmix and 2.5 μl of plasmid carrying the gene of interest were mixed in another tube. The entire reaction mixture was carefully transferred to the bottom of the PCR tube containing the SUB-AMIX TT (50 μl) to create a bilayer preparation. The mixture was then incubated at 26°C for 24 hours. Upon completion of the protein expression reaction, the reaction mixture was gently mixed and used in subsequent experiments such as fluorescence gel scanning or Western blotting.

QUMA-1 detection

Cells were seeded in a glass-bottom 24-well plate. Subsequently, the cells were fixed with 4% paraformaldehyde in DEPC-PBS at room temperature for 15 minutes. After rinsing with DEPC-PBS, the cells were stained with 1 μM QUMA-1 for 20 minutes at 37°C. Coverslips were then mounted with Vectashield mounting medium containing DAPI (Vector Laboratories) and visualized using fluorescence microscopy (ZEISS Axio Observer7).

Western blot (WB)

Proteins were separated by SDS-PAGE and transferred to PVDF membrane. The following antibodies were incubated with membrane: anti-DHX9(1:1000, 18821-1-AP, proteintech), anti-p-eIF2α(1:1000, 9721S, CST), anti-p16 (1:1000, 18769S, CST), anti-mCherry (1:1000, 26765-1-AP, Proteintech), anti-GAPDH (1:5000, 60004-1-Ig, Proteintech). And the secondary antibodies are HRP-conjugated anti-rabbit or anti-mouse (KPL, Inc).

Quantitative real-time PCR

Total RNA was extracted from cells using RNAiso Plus Reagent (9109, Takara) according to manufacturer's instructions. 1 μg of total RNA was reverse-transcribed to cDNA using PrimeScript RT reagent Kit (RR047A, Takara). cDNA was used for real-time PCR using 2×RealStar Green Fast Mixture (Takara). β-Actin was used as internal control for all experiments. The following primers were used for amplification: β-Actin-forward: 5'- CATGTACGTTGCTATCCAGGC-3'; β-Actin-reverse: 5'- CTCCTTAATGTCACGCACGAT-3'; p16-forward: 5'-GGGTTTTCGTGGTTCACATCC-3'; p16-reverse: 5'-CTAGACGCTGGCTCCTCAGTA-3';mCherry-forward: 5'- CATCCTGTCCCCTCAGTTCATG-3'; mCherry-reverse: 5'- GGGGAAGGACAGCTTCAAGTAGTC-3'; GFP-forward: 5'- GTTCAGCGTGAGCGGCGAG-3'; GFP-reverse: 5'-CTGCACGCCGTAGCTCAGG-3'; MMP3-forward: 5'-CAAAACATATTTCTTTGTAGAGGACAA-3'; MMP3-reverse: 5'-TTCAGCTATTTGCTTGGGAAA-3'; IFNβ-forward: 5'-AGGACAGGATGAACTTTGAC-3'; IFNβ-reverse: 5'- TGATAGACATTAGCCAGGAG-3'; IL8-forward: 5'-ACTGAGAGTGATTGAGAGTGGAC-3'; IL8-reverse: 5'- AACCCTCTGCACCCAGTTTTC-3'; IL1α-forward: 5'-AGATGCCTGAGATACCCAAAACC-3'; IL1α-reverse: 5'- CCAAGCACACCCAGTAGTCT-3'; IL1β-forward: 5'-TGCACGCTCCGGGACTCACA-3'; IL1β-reverse: 5'- CATGGAGAACACCACTTGTTGCTCC-3'; CXCL1-forward: 5'-AGCTTGCCTCAATCCTGCATCC-3'; CXCL1-reverse: 5'- TCCTTCAGGAACAGCCACCAGT-3'.

SA-β-Gal staining

The SA-β-Gal staining was performed with the staining kit (C0602, beyotime). Briefly, after washed cells with PBS for 3 times, used stationary liquid fix 15 mins at room temperature. Then wash cells with PBS for 3 times, and stain with the staining buffer for 12 h at 37°C.

G4P-RIP-seq

The RIP was according to the book Methods in Molecular Biology. G4P-stable expressing cells were grown in an appropriate culture medium and induced with DOX. To cross-link the cells, the formaldehyde solution was added to achieve a final concentration of 1% and incubated for 10 minutes at room temperature (RT). The reaction was stopped by adding one-tenth the volume of 2.66 M glycine and incubating for an additional 5 minutes at RT. Then collected cells and centrifugated at 1000 ×g for

5 minutes at 4 °C, and the supernatant was discarded. The cells were washed twice with 1× ice-cold PBS. Next, the cells were resuspended in 4 mL of cell lysis buffer (10 mM Tris-HCl pH 7.4, 10 mM NaCl, 0.5 % IGEPAL,1 mM DTT, 200 units/ml RNase OUT, and EDTA-free Protease Inhibitor Cocktail) and incubated for 15 minutes on ice. The nuclei were sonicated to obtain DNA fragments within the range of 200 to 1000 bp. After the sonication, 250 units/mL of DNase were added to the chromatin and incubated for 30 minutes at 37 °C. In a separate 1.5 ml tube, 20 µl of Flag magnetic beads were added and washed twice with 0.5 ml of NT-2 buffer (50 mM Tris-HCl pH 7.4, 150 mM NaCl, 1 mM MgCl2, 0.05 % IGEPAL).

Centrifuge the cell lysate at 20,000 ×g for 10 minutes at 4 °C. Remove 975 µl of the supernatant and add it to each Flag beads reaction. Take 10% of the cell lysate supernatant and transfer it to a new tube labeled "Input". Incubate all tubes on a rotating wheel for 6 hours at 4 °C. After the overnight incubation, spin down the tubes, place them on a magnetic support over ice, and discard the supernatant. Resuspend each immunoprecipitate in 150 µl of Proteinase K buffer(50 mM Tris-HCl pH 7.4, 150 mM NaCl, 1 mM MgCl2, 0.05 % IGEPAL, 1% SDS, 1.2 mg/ml Proteinase K). For each "Input" sample, add 107 µl of NT-2, 15 µl of 10% SDS, and 18 µl of Proteinase K, reaching a total volume of 150 µl. Incubate all tubes at 55 °C for 30 minutes with shaking to digest the proteins.

Recover the RNA by phenol:chloroform:isoamyl alcohol (125:24:1) and absolute ethanol precipitation. Resuspend the pellets in 20 µl of RNase-free water and place the tube on ice. Treat all the volume of each sample with DNase to remove residual contaminant DNA in the further analysis.

Polysome analysis

Polysome analysis was performed according to the established protocol. Briefly, 10–45% sucrose density gradient was prepared in resolving buffer (containing 100 mM NaCl, 25 mM Tris-Cl, pH 7.4, and 5 mM MgCl2) using a Biocomp gradient maker. Cell samples were treated with 100 µg/ml cycloheximide for 15 minutes at 37°C. Cells were harvested and washed three times with ice-cold PBS supplemented with cycloheximide, followed by resuspension of the cell pellets in 300 µL of lysis buffer (comprising 25 mM Tris-HCl, pH 7.4, 100 mM NaCl, 5 mM MgCl2, 1 mM DTT, 1% sodium deoxycholate, 1% IGEPAL, 40 U/mL RNase inhibitor, 1× EDTA-free complete protease inhibitors, and 100 µg/ml cycloheximide). The cell lysate was centrifuged at

13,000 g for 10 minutes at 4 °C. The supernatant was then transferred to a new pre-chilled 1.5 mL tube. The RNA concentration in the cell lysate was normalized across samples, and equal quantities of RNA were loaded onto each sucrose gradient. The gradients were placed in an SW41 rotor and centrifuged at 36,000 rpm for 3 hours at 4 °C. After centrifugation, the sucrose gradients were unloaded from the ultracentrifuge and fractionated while simultaneously monitoring the optical density at 260 nm using a Biocomp fractionator station.

Puromycin incorporation assay

Cells were treated with 1 µM Puromycin for 20 minutes prior to harvesting. Cells were then lysed using RIPA buffer (comprising 150 mM NaCl, 50 mM Tris, pH 7.4, 0.25% sodium deoxycholate, 1 mM EDTA, and 1% IGEPAL). Proteins were separated by SDS-PAGE and subsequently transferred to PVDF membrane. Before blocking, the membranes were stained with Ponceau-S solution for 5 minutes. The membranes were then incubated with an anti-puromycin antibody (1:1000, ab315887, Abcam) followed by HRP-conjugated anti-rabbit secondary antibodies (KPL, Inc).

Quantification and statistical analysis

The data are presented in a format that clearly depicts the distribution of the data, typically in bar graphs. All elements of the graphs, including the median and error bars, are fully explained in the figure legends. The sample size was not predetermined by any statistical tests. Throughout the figures and corresponding legends, ‘n’ represents the number of independent experiments unless otherwise specified in the figure legend. GraphPad Prism 8 was used for statistical analysis. Results are shown as mean ± SEM and the unpaired Student’s two-tailed t-test or one-way ANOVA was used to determine the statistical significance (**P*<0.05; ***P*<0.01; ****P*<0.001, ****P<0.0001).

References

DOBIN, A., DAVIS, C. A., SCHLESINGER, F., DRENKOW, J., ZALESKI, C., JHA, S., BATUT, P., CHAISSON, M. & GINGERAS, T. R. 2013. STAR: ultrafast universal RNA-seq aligner. *Bioinformatics,* 29**,** 15-21.

HEINZ, S., BENNER, C., SPANN, N., BERTOLINO, E., LIN, Y. C., LASLO, P., CHENG, J. X., MURRE, C., SINGH, H. & GLASS, C. K. 2010. Simple combinations of lineage-determining transcription factors prime cis-regulatory elements required for macrophage and B cell identities. *Mol Cell,* 38**,** 576-89.

LANGMEAD, B., TRAPNELL, C., POP, M. & SALZBERG, S. L. 2009. Ultrafast and memory-efficient alignment of short DNA sequences to the human genome. *Genome Biology,* 10.

LI, F., XING, X., XIAO, Z., XU, G. & YANG, X. 2020. RiboMiner: a toolset for mining multi-dimensional features of the translatome with ribosome profiling data. *BMC Bioinformatics,* 21.

LIAO, Y., SMYTH, G. K. & SHI, W. 2014. featureCounts: an efficient general purpose program for assigning sequence reads to genomic features. *Bioinformatics,* 30**,** 923-930.

LOVE, M. I., HUBER, W. & ANDERS, S. 2014. Moderated estimation of fold change and dispersion for RNA-seq data with DESeq2. *Genome Biology,* 15.

MARTIN, M. 2011. Cutadapt removes adapter sequences from high-throughput sequencing reads. *EMBnet.journal,* 17.

XIAO, Z., HUANG, R., XING, X., CHEN, Y., DENG, H. & YANG, X. 2018. De novo annotation and characterization of the translatome with ribosome profiling data. *Nucleic Acids Research,* 46**,** e61-e61.

XIAO, Z., ZOU, Q., LIU, Y. & YANG, X. 2016. Genome-wide assessment of differential translations with ribosome profiling data. *Nature Communications,* 7.

XU, W., LIU, C., DENG, B., LIN, P., SUN, Z., LIU, A., XUAN, J., LI, Y., ZHOU, K., ZHANG, X., HUANG, Q., ZHOU, H., HE, Q., LI, B., QU, L. & YANG, J. 2022. TP53-inducible putative long noncoding RNAs encode functional polypeptides that suppress cell proliferation. *Genome Research,* 32**,** 1026-1041.

YAN, Y., TANG, R., LI, B., CHENG, L., YE, S., YANG, T., HAN, Y.-C., LIU, C., DONG, Y., QU, L.-H., LUI, K. O., YANG, J.-H. & HUANG, Z.-P. 2021. The cardiac translational landscape reveals that micropeptides are new players involved in cardiomyocyte hypertrophy. *Molecular Therapy,* 29**,** 2253-2267.
